# Decomposition analysis on the excitation behaviors of thiazolothiazole (TTz)-based dyes *via* the time-dependent dielectric density functional theory approach†

**DOI:** 10.1039/d2ra06454e

**Published:** 2022-12-02

**Authors:** Takumi Naito, Yukiumi Kita, Tomomi Shimazaki, Masanori Tachikawa

**Affiliations:** Graduate School of Nanobioscience, Yokohama City University 22-2 Seto, Kanazawa-ku Yokohama 236-0026 Japan tshima@yokohama-cu.ac.jp

## Abstract

Thiazolothiazole (TTz)-based materials have been attracting much attention because of their widespread applications. In this paper, we discuss the excited electronic behaviors of asymmetric TTz dyes in solvents based on the time-dependent dielectric density functional theory method. Based on dipole moment and charge distribution (population) analyses, we discuss large intramolecular electron transfers, which are triggered by photon excitations, toward the acceptor part of dyes. In addition, we explore the contributions of geometrical changes and solvent reorientations (reorganizations) to the solvatofluorochromic phenomena based on a decomposition technique. The decomposition analysis shows that the solvent reorientation effect mainly contributes to changes in the fluorescent spectra in response to solvents.

## Abstract 2

Decomposition analysis shows that the solvent reorientation effect causes solvatofluorochromism of TTz dyes.

## Graphical abstract

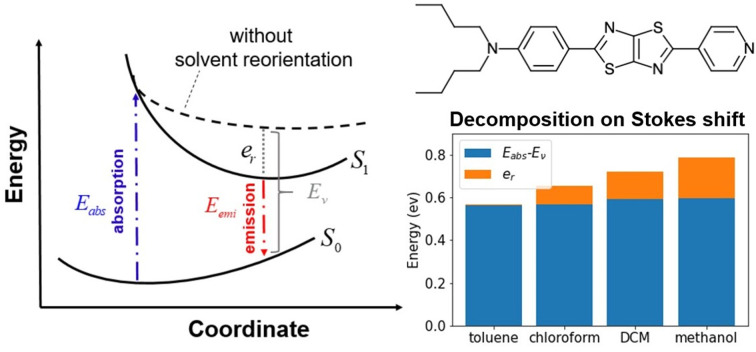

## 1. Introduction

Organic materials with the thiazolothiazole (TTz) molecular backbone have been widely employed in various applications, such as organic electronics, displays, photonics, and fluorescent sensors.^1–9^ The rigid and coplanar structure of TTz is suitable for extending π-conjugated systems of organic materials with high carrier mobility. For example, organic photocells employ TTz units in polymer materials, which are effective for not only developing highly efficient devices but also improving device stability and manufacturing reproducibility.^10,11^ In such devices, TTz is used as rigid π-bridge blocks to control the crystallinity and morphology of organic compounds. In addition to applications as a polymer building block, TTz-based (small) molecules were recently proposed as acceptor materials to replace fullerene derivatives.^12^ The non-fullerene organic photocell devices have been actively studied because of their superior advances in power conversion efficiency.^13–16^ The TTz molecular framework is promising to develop non-fullerene acceptor materials for organic photocells. TTz-based dyes are intensively studied for bioimaging applications. For example, Kumar *et al.* reported a fluorescent imaging technique for cells by using a TTz-based dye with pyrene and pyridine moieties serving as donor and acceptor parts, respectively.^17^ Sayresmith *et al.* developed TTz-bridged dye sensors, which have an electron-donor part (*N*,*N*-dibutylaniline or *N*,*N*-diphenylaniline) and an electron-acceptor part (pyridine, benzoic acid, or carboxaldehyde) by using a simple one-pot and single-step synthesis techniques, and they reported that the strong solvatofluorochromic characteristics of these dyes can be effectively utilized for imaging cell membranes.^18^

To develop fluorescent dyes, the molecular design based on donor–π–acceptor, with TTz as the π-bridge, is frequently employed. The electron-donating and -withdrawing (push–pull) functional groups are introduced at the ends of the TTz-bridge, and they serve as the donor and acceptor parts, respectively, in the dye. Such an asymmetric molecular design from the viewpoint of TTz is essential to control intermolecular electron transfers toward the acceptor part in excited dyes. In this paper, we discuss the intermolecular electron transfer process of excited asymmetric TTz dyes. We focus on the solvatofluorochromic phenomena because of its important applications such as cellular imaging to investigate the response of dyes to dielectric environments.

During photon absorption, a dye is vertically excited from the stable molecular conformation in the ground state. In contrast, in the fluorescent state (emission), the conformation of the dye is relaxed under the excited state to emit a photon. In addition to the structural (conformational) relaxation, the reorientation (reorganization) of solvent molecules in response to the excited dye should be taken into account to consider the fluorescent spectra.^19,20^ To discuss these effects, we performed a decomposition analysis, where the contributions from the conformational changes of the dye and solvent reorientations were distinguished by using density functional theory (DFT) calculation techniques.

In this study, we employed the dielectric-dependent approach^21^ in the time-dependent DFT framework^22–27^ to examine the excitation behaviors of dyes. The basic concept of the dielectric DFT approach was derived from the simplified dielectric function model,^21^ which is closely related to the GW method.^28^ Thus, the dielectric-dependent approach has been verified mainly for inorganic bulk materials and used to investigate their material properties.^21,29–46^ In addition, applications of the dielectric DFT method toward molecular systems are also developing in recent years. We previously reported that the dielectric-dependent DFT method can give good descriptions of the electronic structures of molecules in the ground and excited states.^47–49^ Conversely, this paper will focus more on TTz excitation behaviors with the structural change and solvent reorientation effects.

In the next section, we briefly explain the dielectric DFT approach. In Section 3, we present the calculation results and discussions, including the intermolecular electron (charge) transfer process of asymmetric TTz dyes, with emphasis on the structural change and solvent reorientation effects. The summary is provided in Section 4.

## 2. Calculation method

The dielectric exchange screening term can be derived from the simplified semiconductor dielectric function of *ε*(*k*) = 1 + [(*ε*_s_ − 1)^−1^ + *β*(*k*^2^/*k*_TF_^2^)]^−1^ with dielectric constant *ε*_s_.^21^ Here, *k* is the wavenumber; *k*_TF_ is the Thomas–Fermi wavenumber; *β* is a constant parameter introduced by Bechstedt *et al.*^50^ In this study, we applied *β* = 2.5 for all the molecules.^49^ From the model dielectric function, we can obtain the following screened exchange potential.1
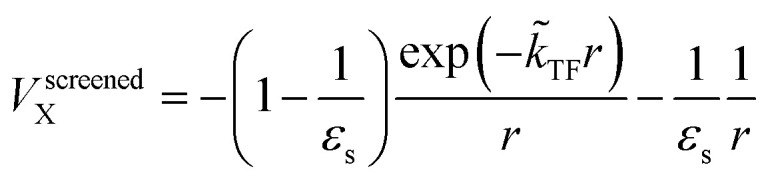
Here, 
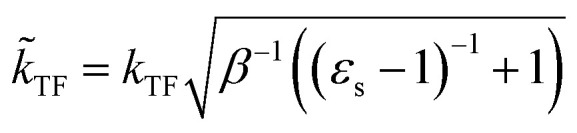
. The inverse Fourier transformation of *V*^screened^_X_ = −(2π)^−3^∫4π*k*^−2^*ε*^−1^(*k*)exp(*i**k***·***r***)d***k*** is employed to derive the equation. The second term in eqn (1) describes the incomplete screening in a semiconductor, which is proportional to the inverse of the dielectric constant *ε*_s_. For *ε* = 1, the screened exchange potential represents a bare interaction between an electron and exchange hole in the Hartree–Fock (HF) approximation. Conversely, it reduces to a Thomas–Fermi (Yukawa)-type screened potential in the case of metals (*ε* → ∞), and complete screening is achieved. To efficiently handle the short-range term in the Gaussian basis set framework, we can employ erfc(2*k̃*_TF_*r*/3)/*r* instead of exp(−*k̃*_TF_*r*)/*r* because of their similar behaviors.^21^ The Coulomb hole (COH) interactions in the COHSEX approximation of the GW method can be considered by using the simplified model dielectric function.^31^ The concept of the dielectric-screening potential can then be incorporated into the conventional hybrid DFT framework as follows:2
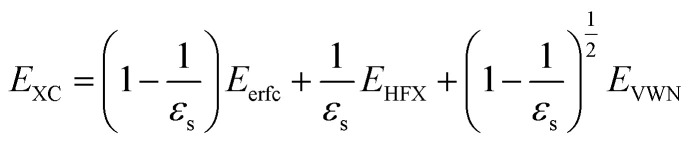
Here, *E*_erfc_ is the erfc-based short-range functional, and *E*_HFX_ is the Hartree–Fock (HF) exchange functional, which corresponds to the first and second terms of eqn (1), respectively. *E*_VWN_ is the Vosko–Wilk–Nusair DFT correlation functional.^51^ The nonlocal behavior of the erfc-based short-range term is small, and therefore, the following local potential approximation can work well.3
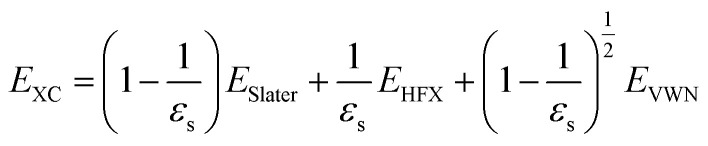
Here, *E*_Slater_ is the local Slater-type DFT exchange functional.^52^ To determine the dielectric constant *ε*_s_, we incorporate the following random-phase approximation^53,54^ into the self-consistent field loop of the quantum chemistry method.^29,31^4
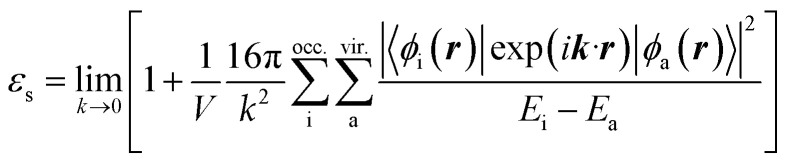
Here, the indices i and a represent the occupied and virtual (unoccupied) orbitals, respectively. *E*_i_ and *E*_a_ are the energies of the occupied and virtual orbitals *ϕ*_i_ and *ϕ*_a_, respectively. We use the value 0.001 a.u. to determine the boundary of the molecular volume *V*. The isotropic average in the limiting case *k* → 0 is used to evaluate the dielectric constant.

**Fig. 1:**
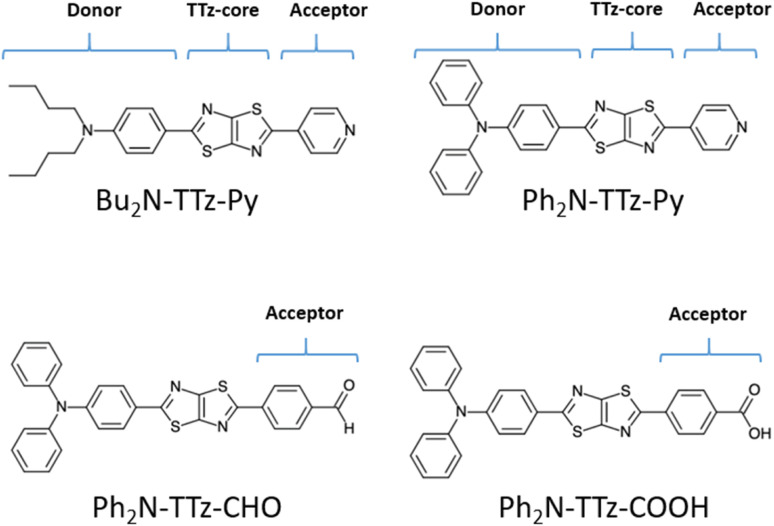
Chemical structure of asymmetric TTz dyes. The definitions of the donor, TTz-core, and acceptor parts used in the charge (electron) distribution analysis are also provided.

We discuss the absorption and emission spectra of asymmetric TTz dyes obtained by the dielectric-dependent approach, and we compare our results with those obtained using B3LYP and HandH methods with the nonlocal exchange ratios of 20% and 50%, respectively.^55,56^ In this study, we used Gaussian16 program for geometry optimization and excited electronic structure calculations with the 6-31+g* basis set.^57^ We estimated the dielectric constants of dyes by the self-consistent technique based on eqn (4) using a dynamic-language-based quantum chemistry tool.^58,59^ This study focused on asymmetric TTz dyes synthesized by Sayresmith *et al.*, which are shown in Fig. 1.^18^ The self-consistent method using eqn (2) provides the constants as 2.80, 3.13, 3.20, and 3.14 are calculated for Bu_2_N-TTz-Py, Py_2_N-TTz-Py, Py_2_N-TTz-CHO, and Py_2_N-TTz-COOH, respectively, whereas eqn (3) gives slightly different values of 2.78, 3.11, 3.18, and 3.11, respectively, for these dyes. We used these dielectric constants for excitation energy calculations. In this study, the polarizable continuum model using the integral equation formalism was adopted to handle the solvent effects.^60^ The same calculation procedures based on the time-dependent DFT method were employed in previous studies.^49^

## 3. Results and discussions

### 3.1 Absorption and emission spectra calculations

The absorption energies obtained from first-principles quantum chemistry methods are summarized in Table 1, where experimental results are also presented for comparison.^18^ Previous studies have shown that the absorption energies of TTz dyes are not strongly affected by the solvent effect unlike the emission spectra.^18^ This tendency can be reproduced in the quantum chemistry calculations. In contrast, the B3LYP (HandH) method tends to underestimate (overestimate) the experimental absorption energies of TTz dyes, though the dielectric-dependent approach yields better results. For example, the B3LYP (HandH) method gives absorption spectra of 2.55 (3.27) eV for Bu_2_N-TTz-Py in dichloromethane (DCM), whereas the corresponding experimental result is 2.89 eV. The dielectric-dependent method using eqn (2) provides an absorption energy of 2.92 eV. In the ESI,† we showed calculation results when basis sets are changed. We summarized calculation results using the CAM-B3LYP^61^ and M06-2X^62^ methods, which tend to slightly overestimate absorption energies for these TTz dyes. The HF exchange ratio is automatically tuned by using the self-consistent scheme based on eqn (4) in the dielectric-dependent approach. This characteristic behavior may be useful to describe the electronic structure of dyes in the excited state.

**Table 1:** Absorption energies [eV] of asymmetric TTz dyes in solvents

	B3LYP	HandH	B3LYP-CAM	M06-2X	Eqn (2)	Eqn (3)	Exp.
Bu_2_N-TTz-Py							
Toluene	2.61	3.30	3.25	3.25	2.94	3.01	—
Chloroform	2.57	3.28	3.24	3.23	2.92	2.99	2.88
DCM	2.55	3.27	3.23	3.22	2.92	2.98	2.89
Methanol	2.55	3.28	3.23	3.22	2.93	2.98	2.93

Ph_2_N-TTz-Py							
Toluene	2.50	3.24	3.24	3.20	2.85	2.83	2.96
Chloroform	2.49	3.23	3.23	3.20	2.85	2.82	2.93
DCM	2.48	3.23	3.23	3.20	2.88	2.82	2.94
Methanol	2.48	3.24	3.24	3.21	2.91	2.83	—

Ph_2_N-TTz-CHO							
Toluene	2.30	3.13	3.15	3.12	2.75	2.66	2.92
Chloroform	2.28	3.12	3.15	3.12	2.76	2.64	—
DCM	2.27	3.12	3.15	3.12	2.77	2.64	2.86
Methanol	2.27	3.13	3.16	3.13	2.79	2.64	—

The theoretical and experimental emission energies of asymmetric TTz molecules are listed in Table 2. To obtain the emission (fluorescence) spectra, the geometrical optimization calculations under the excited electronic state were performed by considering the solvent effect by adopting the time-dependent DFT method. Unlike the absorption spectra, the theoretical and experimental emission energies are strongly affected by solvents. For example, eqn (3) yields energy values of 2.48 and 2.35 eV for Ph_2_N-TTz-Py in toluene and DCM, respectively, while the corresponding experimental results are 2.57 and 2.31 eV, respectively. The dielectric-dependent approach can describe well the electronic structure of asymmetric TTz dyes in the excited state. Hence, we focus on calculations and discussions based on the dielectric-dependent method in the following sections.

**Table 2:** Emission energies [eV] of asymmetric TTz dyes in solvents

	B3LYP	HandH	CAM-B3LYP	M06-2X	Eqn (2)	Eqn (3)	Exp.
Bu_2_N-TTz-Py							
Toluene	2.40	2.75	2.66	2.67	2.38	2.64	—
Chloroform	2.29	2.64	2.55	2.56	2.27	2.52	2.33
DCM	2.23	2.58	2.48	2.49	2.20	2.46	2.41
Methanol	2.17	2.51	2.42	2.43	2.14	2.40	2.20

Ph_2_N-TTz-Py							
Toluene	2.12	2.70	2.64	2.64	2.31	2.48	2.57
Chloroform	2.11	2.60	2.54	2.55	2.22	2.40	2.39
DCM	2.09	2.55	2.49	2.49	2.16	2.35	2.31
Methanol	2.06	2.50	2.43	2.44	2.11	2.30	—

Ph_2_N-TTz-CHO							
Toluene	2.00	2.57	2.52	2.52	2.19	2.33	2.48
Chloroform	1.96	2.47	2.42	2.43	2.09	2.24	—
DCM	1.92	2.41	2.36	2.37	2.03	2.18	2.21
Methanol	1.89	2.35	2.30	2.32	1.98	2.13	—

### 3.2 Ph_2_N-TTz-COOH monomers and dimers in solvents

**Fig. 2:**
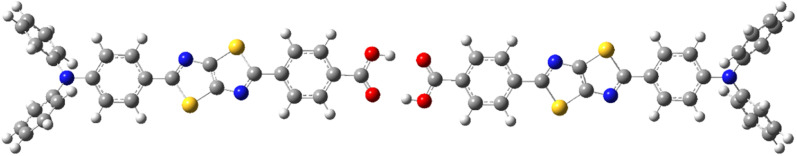
Optimized dimer structure of Ph_2_N-TTz-COOH obtained from the dielectric-dependent density functional theory (DFT) method with eqn (2).

This section provides the calculation results of Ph_2_N-TTz-COOH considering both monomers and dimers. Ph_2_N-TTz-COOH with carboxyl group may take a dimmer structure in low-polarity solvents. Fig. 2 shows the ground state Py_2_N-TTz-COOH dimer structure in DCM obtained from the optimization calculation using the dielectric-dependent DFT method with eqn (2). The absorption and emission excitation energies of monomers and dimers in solvents are summarized in Table 3. From these calculations, we can confirm that small differences exist between monomer and dimer spectra. For example, the absorption excitation energies of monomer and dimer TTz dyes in toluene were evaluated as 2.82 and 2.81 eV, respectively, using eqn (2), and the experimental result was 2.93 eV. Further, the emission spectra of monomer and dimer TTz dyes in toluene were calculated as 2.23 and 2.22 eV, respectively, and the experimentally measured value was 2.55 eV. Thus, we will discuss monomers for Ph_2_N-TTz-COOH in the subsequent sections.

**Table 3:** Absorption and emission energies [eV] of Ph_2_N-TTz-COOH

	Toluene	Chloroform	Dichloromethane	Methanol
**Absorption**				
Exp.	2.93	2.92	2.93	3.00

**Monomer**				
Eqn (2)	2.82	2.84	2.84	2.86
Eqn (3)	2.78	2.75	2.74	2.75

**Dimer**				
Eqn (2)	2.81	2.81	2.82	2.83
Eqn (3)	2.73	2.71	2.70	2.70

**Emission**				
Exp.	2.55	2.26	2.37	2.32

**Monomer**				
Eqn (2)	2.23	2.13	2.08	2.03
Eqn (3)	2.39	2.31	2.26	2.21

**Dimer**				
Eqn (2)	2.22	2.13	2.07	2.02
Eqn (3)	2.37	2.13	2.07	2.02

### 3.3 Dipole moment and charge (electron) distribution analyses

**Fig. 3:**
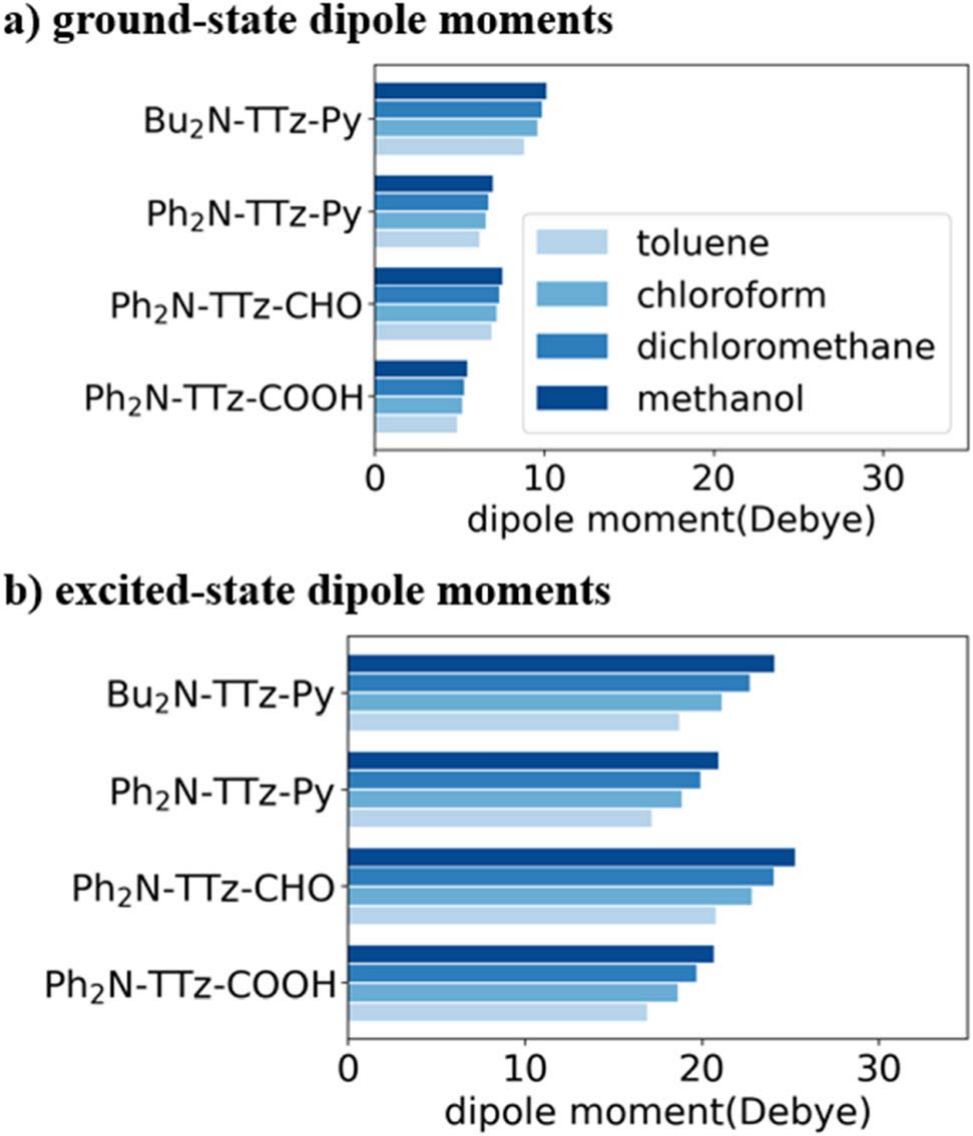
Dipole moment [Debye] of asymmetric TTz dyes in (a) the ground and (b) excited states. The optimized structures of the excited dyes are employed to obtain the dipole moments in the excited state. These dipole moments increase greatly because of charge (electron) transfers in the dyes, and they are also affected by the solvents around the dyes.

To investigate the excited-state behaviors of asymmetric TTz dyes, we first focused on their dipole moments in the ground and excited states in solvents, as shown in Fig. 3, based on the dielectric DFT method using eqn (2). These calculation results show that molecular excitations can induce significant modifications in the dipole moments. The dipole moments in the excited state are several times those in the ground state. For example, the dipole moments of Ph_2_N-TTz-CHO in the ground and excited states in toluene are obtained as 6.88 and 20.77 debye, respectively. We summarized the detailed data in the ESI.† In addition, the dipole moments in the excited state are largely affected by surrounding solvents; for example, the dipole moments of Ph_2_N-TTz-Py in the excited state in toluene, chloroform, and methanol are 17.14, 18.86, and 20.91 debye, respectively. Thus, the dipole moment analysis suggests that electronic structures of dyes are modified by molecular excitations and solvents.

**Fig. 4:**
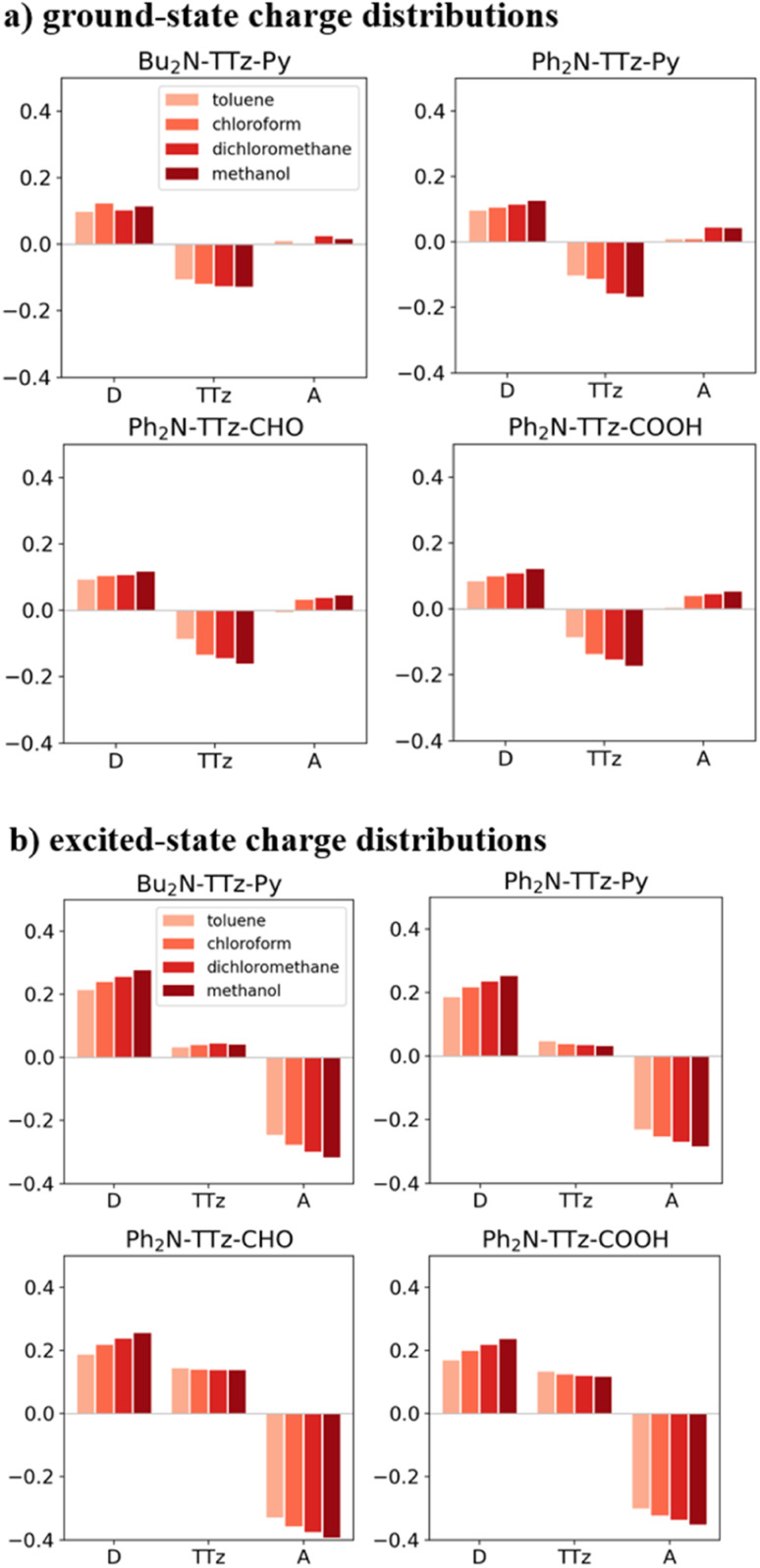
Charge distributions of the TTz dyes in the (a) ground and (b) excited states. The definitions of acceptor (A), TTz, and donor (D) regions are depicted in Fig. 1. Here, the optimized structures of the excited state are employed to obtain the charge distributions in the excited state. The intermolecular electron transfers toward the acceptor region, which are induced by molecular excitations, drive large changes in the charge distributions in the dyes.

**Fig. 5:**
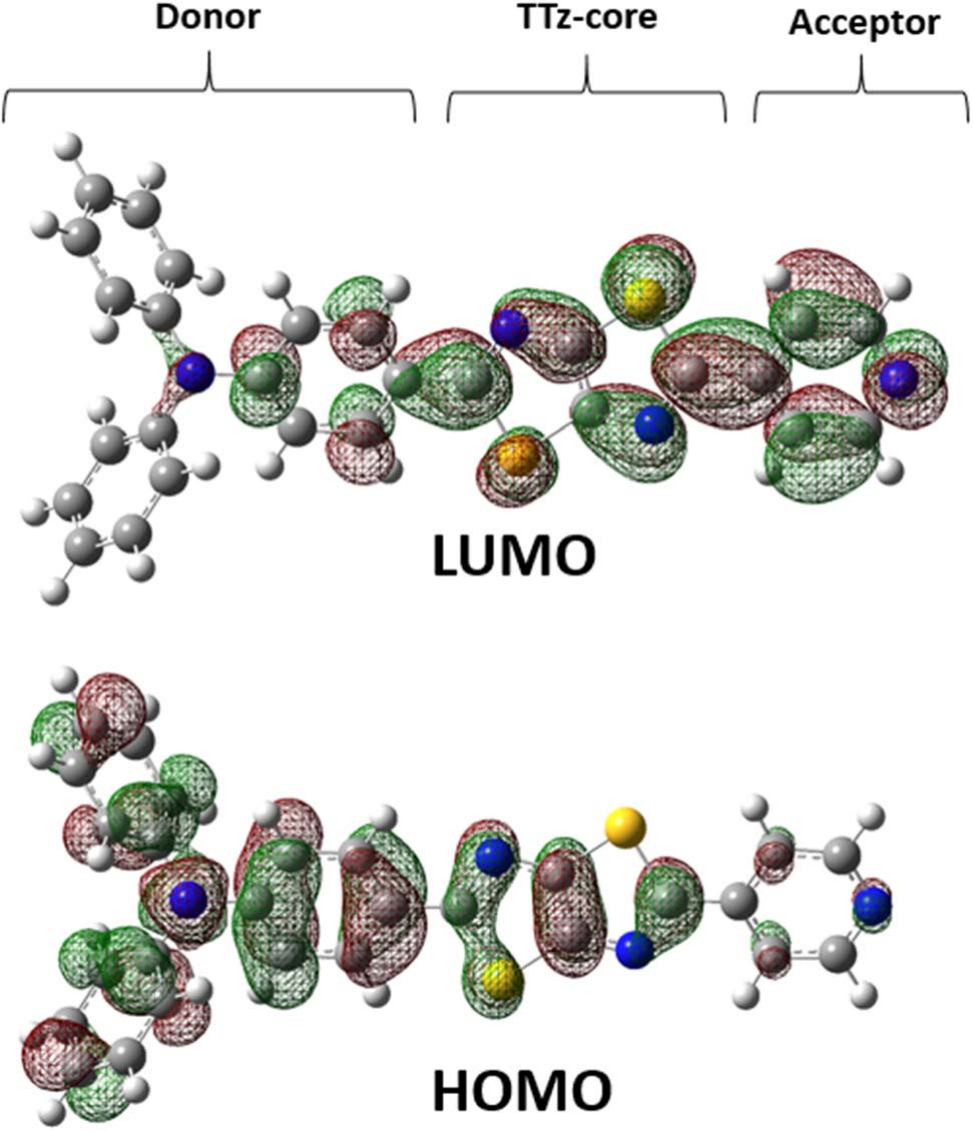
Highest occupied molecular orbital (HOMO) and lowest unoccupied molecular orbital (LUMO) molecular orbitals of Ph_2_N-TTz-Py in dichloromethane.

Next, we discuss the charge (electron) distributions in the dyes in the ground and excited states. For this purpose, we considered molecules to be composed of three parts, namely, the donor, TTz-core, and acceptor regions, as shown in Fig. 1. Fig. 4 summarizes the charge (electron) distributions aggregated in these parts (moieties) in the ground and excited states. We employed the Mulliken-based population analysis to obtain the charge distributions in the dyes. From these calculations, we observe that molecular excitations can also induce large changes on the charge (electron) distributions; each part in the excited state has larger negative or positive partial charges compared with those in the ground state. For example, the donor part of Bu_2_N-TTz-Py has a positive charge of about 0.1 a.u. in methanol under the ground state, and the charge increases greatly to around 0.4 a.u. in the excited state. The acceptor part has almost no charge in the ground sate, but it is negatively charged (about −0.2 a.u.) in the excited state. The partial charge on the TTz-core part increases to about −0.2 from −0.1 a.u. in the response to changes in charge distribution. In particular, the behavior of the acceptor part may be interesting; the polarization of the dye in the ground state occurs only in the donor and TTz-core regions, but it spreads throughout the molecule, including the acceptor region, in the excited state. These behaviors can be observed in the case of other asymmetric TTz dyes. These phenomena may be explained based on the shapes of the highest occupied molecular orbital (HOMO) and lowest unoccupied molecular orbital (LUMO). Fig. 5 shows the molecular orbitals of Ph_2_N-TTz-Py, where HOMO has a small participation with regard to the acceptor part, but LUMO has a much larger contribution. In asymmetric TTz dyes, an excited electron mainly transitions from the HOMO to the LUMO. For example, the largest configurational interaction (CI) coefficient of 0.67 is calculated for the transition from the HOMO to the LUMO for Bu_2_N-TTz-Py in toluene for photon absorption. Other cases are summarized in the ESI.† Such an electron transition drives a large intermolecular electron transfer toward the acceptor part in dyes. In addition, the solvent effect influences electron distributions in the excited state. Thus, solvent molecules can affect the electron transfer process between acceptor and donor parts. In the next section, we discuss the solvent effect on asymmetric TTz dyes with focus on the geometrical changes and solvent reorientations (reorganizations) induced by molecular excitations.

### 3.4 Decomposition analysis on the solvatofluorochromism

**Fig. 6:**
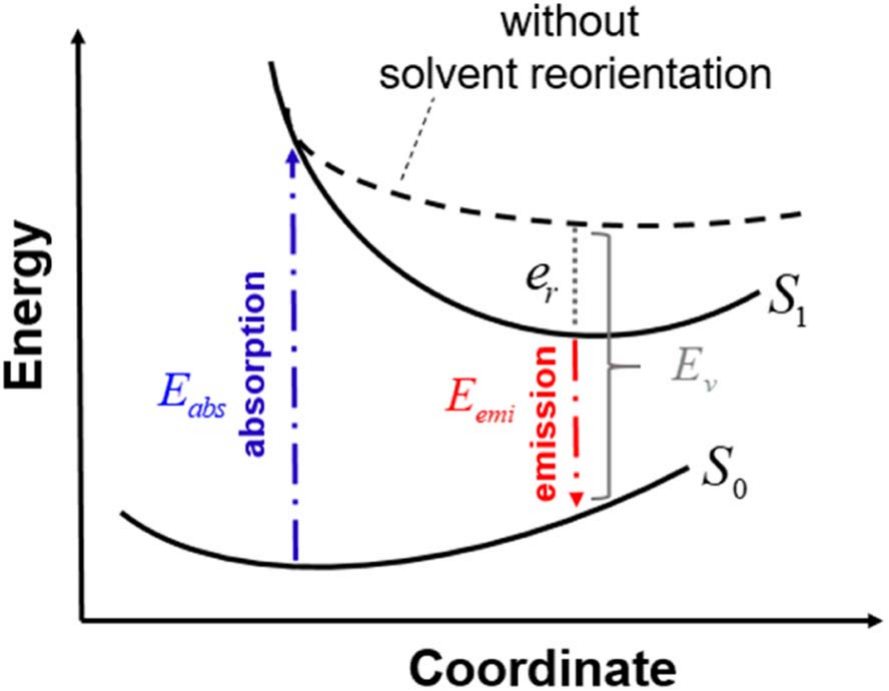
Schematic diagram to explain the decomposition analysis to obtain the contributions of geometrical changes and solvent reorientations (reorganization) induced by molecular excitation. Here, S_0_ and S_1_ represent the potential energy surfaces of an asymmetric TTz dye in the ground and excited states, respectively. The energy surface S_1_ includes the solvent reorientation effect. *E*_abs_ and *E*_emi_ are the absorption and emission energies, respectively, and the difference between them gives the Stokes shift. The dashed line represents excitation energies without the solvent reorientation effect, which are difficult to obtain experimentally. *E*_v_ is the vertical excitation energy without the reorientation effect. In this study, we evaluated the solvent reorientation contribution on the solvatofluorochromic effect of dye using the expression *e*_r_ = *E*_v_ − *E*_emi_.

In this section, we discuss the Stokes shift property, which can be evaluated from the difference between the absorption and emission excitation energies of the dye. When asymmetric TTz dyes absorb photons, their electronic structures are greatly modified by the intermolecular electron (charge) transfer process, as discussed in the previous sections. Consequently, the geometric conformation of the excited dye relaxes toward a more energetically stable one under the excited state. Such conformational (geometrical structure) changes can strongly influence the Stokes shift. On the other hand, it is essential to consider the interactions between dyes and solvents when dealing with molecular emissions. Solvent molecules change their orientations depending on the excited electronic structure of dyes; this phenomenon is known as the solvent reorientation (reorganization) effect. The Stokes shifts of dyes are also affected by the solvent reorientation effect. In this section, we divide the Stokes shift into parts contributed by the geometrical change in the dye and solvent reorientation. Fig. 6 explains the decomposition technique, where *E*_abs_ and *E*_emi_ are the absorption and emission energies of asymmetric TTz dyes in solvents, respectively. *E*_emi_ is obtained from the time-dependent DFT method by executing geometric optimization in the excited state with the solvent reorientation effect. Conversely, the value of *E*_v_ is the vertical excitation energy without the solvent reorientation effect, though the optimized molecular coordinate in the excited state is employed. *E*_v_ is usually difficult to observe experimentally, and therefore, it may be a virtual computed quantity. However, we can estimate the solvent reorientation effect of *e*_r_ from the difference between *E*_v_ and *E*_emi_. From these relationships, the following decomposition analysis can be employed to investigate the solvatofluorochromic phenomena of asymmetric TTz dyes.Stokes shift = *E*_abs_ − *E*_emi_ = *E*_abs_ − (*E*_v_ − *e*_r_) = (*E*_abs_ − *E*_v_) + *e*_r_Here, the first term represents the contribution of geometrical changes in the dye induced by molecular excitation, and the second term describes the reorientation effect of solvents.

**Fig. 7:**
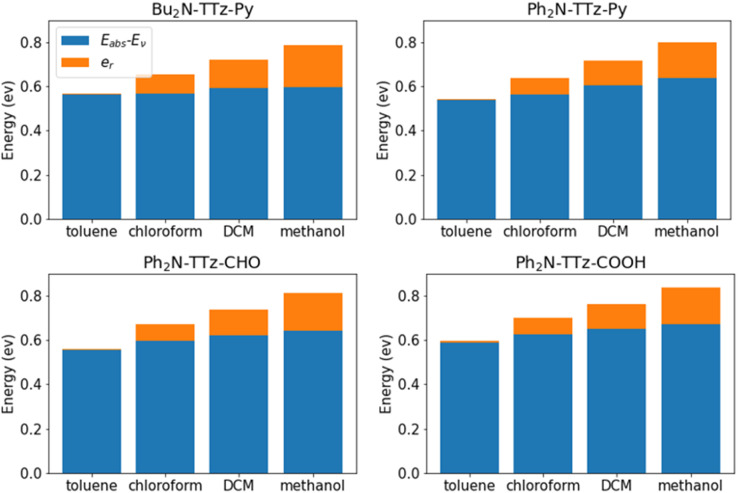
Contributions of the geometrical changes and solvent reorientation to the Stokes shifts of asymmetric TTz dyes. Although geometrical changes have a large impact on the Stokes shift, the solvent reorientation has a greater contribution to the response to solvents. The solvatofluorochromic phenomena are observed as spectral changes depending on the solvents, and therefore, the reorientation effect of the solvent molecules around the dyes mainly causes the solvatofluorochromism of asymmetric TTz dyes.

We analyzed the excited state behaviors of asymmetric TTz dyes based on the decomposition approach. Fig. 7 presents the contributions of the change in molecular geometry (*E*_abs_ − *E*_v_) and solvent reorientation (*e*_r_), which are depicted in blue and orange, respectively. We also summarized these details in the ESI.† These results show that the changes in the molecular geometry have a greater influence on the Stokes shift, especially for low-polarity solvents such as toluene. For example, the conformational change contribution of Bu_2_N-TTz-Py in toluene is 0.56 eV for a Stokes shift of 0.57 eV. For Ph_2_N-TTz-Py in methanol, the contribution of conformational change is 0.64 eV for a Stokes shift of 0.80 eV. In these cases, the conformational change contributions have high ratios of 98.2% and 80.0%. Thus, the conformational changes induced by the excitations of dyes have the largest impact on the Stokes shift. Conversely, the conformational change contributions of dyes themselves undergo little modifications even if the solvents are changed. From the viewpoint of response to solvents, the solvent reorientation contribution is more important. For Bu_2_N-TTz-Py in toluene, the reorientation effect is 0.01 eV, but it changes to 0.09, 0.13, and 0.19 eV when the solvents used are chloroform, dichloromethane, and methanol, respectively. These tendencies can be observed in the case of other dyes as well. The solvatofluorochromism is considered as changes in the emission spectra of dye in response to changes in solvents. Thus, we can conclude that the reorientation (reorganization) effect mainly contributes to solvatofluorochromic phenomena of asymmetric TTz dyes.

**Fig. 8:**
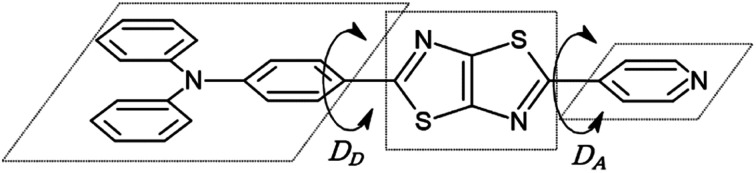
Dihedral angles *D*_A_ (*D*_D_) between the TTz core and acceptor (donor) parts.

Finally, we discuss structural changes induced by the excitation of dyes in solvents. In the ground state, the molecular structures of asymmetric TTz dyes are slightly twisted. To analyze the twists in the molecules, we focused on the (dihedral) angle *D*_A_ (*D*_D_) between the TTz-core part and the acceptor (donor) part, as shown in Fig. 8. In this paper, we simply discuss only the absolute values of the dihedral angles because of the high molecular symmetries. The dihedral angles of TTz molecules in the ground and excited states are summarized in Table 4. For example, the twisted angle *D*_A_ (*D*_D_) is 15.31° (18.17°) for the ground state Ph_2_N-TTz-Py in toluene. The *D*_A_ angles are 15.96°, 20.05°, and 21.06° in chloroform, dichloromethane, and methanol, respectively. The twists in asymmetric TTz molecules change depending on the solvents. In contrast, these twists are released in the excited dyes. That is, asymmetric TTz dyes contain more planar structures because of molecular excitations. For example, *D*_A_ angles in the excited state Ph_2_N-TTz-Py in toluene and methanol are 0.22° and 0.13°, respectively. Changes from twisted structures to planar structures in the excited state have been confirmed for other TTz dyes. These phenomena may be explained by the molecular orbitals depicted in Fig. 5. Although the HOMO mainly participates in the TTz core and acceptor regions, the LUMO is more widely spread throughout the molecule, and therefore, the planar structures of dyes in the excited state may become more stable because of extended conjugated systems. We also observe that the planar structures of excited dyes are influenced when solvents are changed, but these influences are not very large. The TTz-based dyes have a rigid molecular skeleton (structure), and therefore, large conformational (geometrical) changes may be prevented in the excited state. Thus, the solvent reorientation effect may mainly contribute to the solvatofluorochromic phenomena because of the small conformational changes in asymmetric TTz dyes.

**Table 4:** Dihedral (twist) angles [°] of asymmetric TTz dyes in the ground and excited state

	Ground-state		Excited-state	
	*D*_A_	*D*_D_	*D*_A_	*D*_D_
Bu_2_N-TTz-Py				
Hexane	15.50	14.61	0.04	0.45
Chloroform	15.24	13.99	0.31	0.61
Dichloromethane	18.26	14.65	0.74	0.36
Methanol	18.19	16.14	0.08	0.13

Ph_2_N-TTz-Py				
Toluene	15.31	18.17	0.22	0.27
Chloroform	15.96	18.84	0.26	0.43
Dichloromethane	20.05	18.89	0.18	0.67
Methanol	21.06	20.90	0.13	0.88

Ph_2_N-TTz-CHO				
Toluene	17.83	17.77	0.05	0.69
Chloroform	20.85	18.52	0.06	0.99
Dichloromethane	21.91	19.09	0.11	1.07
Methanol	22.94	20.04	0.19	1.18

Ph_2_N-TTz-COOH				
Toluene	19.52	18.50	0.47	0.37
Chloroform	21.70	18.85	0.41	0.70
Dichloromethane	22.62	19.60	0.47	0.78
Methanol	23.52	20.91	0.43	0.93

## 4. Summary

In this paper, we discussed the excited state of asymmetric TTz dyes based on the time-dependent dielectric DFT approach, focusing on the solvatofluorochromic effect. We showed that the results obtained by the dielectric-dependent approach are in good agreement with experimental results related to the absorption and emission (fluorescence) spectra. The dipole moment analysis was performed to investigate the behaviors of the excited dyes, where the dipole moments in the excited state are much larger than those in the ground state. In addition, we evaluated the charge distributions in the ground and excited states of the dyes. In the ground state, the TTz core and donor parts have positive and negative charges, respectively, and there are few charges on the acceptor parts. In contrast, in the excited states of dyes, the acceptor parts can have large negative charges because of intramolecular electron transfers. Thus, the electron transfer process drives large electronic structure changes in excited asymmetric dyes.

To more precisely investigate the solvatofluorochromism of TTz dyes, we performed the decomposition analysis where the Stokes shifts are divided into the contributions arising from geometrical changes and solvent reorientation. The decomposition analysis showed that geometrical changes induced by photon absorptions have the largest impact on the Stokes shifts of asymmetric TTz dyes. Conversely, we also showed that the solvent reorientation effect can have a larger influence on spectral changes in response to solvents. The solvatofluorochromic phenomenon is observed as changes in the emission spectra in response to solvents. Thus, the strong solvatofluorochromic phenomena of asymmetric TTz dyes are caused by the solvent reorientation effect. The rigid molecular structures of TTz-based dyes may prevent large geometrical changes in the excited state, and therefore, the contribution of geometrical changes may become less important.

Several fluorescent dyes have been developed for visualizing intracellular membranes and β-amyloid aggregations.^63,64^ In order to design novel dyes for bioimaging, TTz will play important roles as a molecular backboned. On the other hand, TTz-containing materials and molecules are actively studied especially for dye-sensitized solar cells (DSSCs) and organic photocells.^65,66^ Those studies show strong potential of TTz for applications on DSSCs. In this paper, we discussed the excited electronic structure of functional dyes using several analysis techniques. These analysis results will be useful to develop various types of dye-based materials.

## Conflicts of interest

There are no conflicts to declare.

## Supplementary Material

RA-012-D2RA06454E-s001
