# Supplementary material for: Decomposition analysis on the excitation behaviors of thiazolothiazole (TTz)-based dyes via the time-dependent dielectric density functional theory approach
Source: RSC Adv. 2022 Dec 2;12(53):34685–93. doi: 10.1039/d2ra06454e (PMC9717578; doi:10.1039/d2ra06454e)
Supplement: RA-012-D2RA06454E-s001 [file RA-012-D2RA06454E-s001.pdf]

Electronic Supplementary Material (ESI) for RSC Advances.
This journal is © The Royal Society of Chemistry 2022

Supporting Information

Decomposition analysis on the excitation behaviors of thiazolothiazole (TTz)-based dyes via the time-dependent dielectric density functional theory approach

Takumi Naitou, Yukiumi Kita, Tomomi Shimazaki and Masanori Tachikawa

Graduate School of Nanobioscience, Yokohama City University,

22-2 Seto, Kanazawa-ku, Yokohama 236-0026, Japan

Table S1. Absorption spectra changes according to basis sets. Here, the results of Eq. (2) are obtained by using the same dielectric constants described in the manuscript.

	3-21G	6-31G	6-31G*
**B3LYP**			
Bu_2N-TTz-Py in toluene	2.62	2.56	2.67
Bu_2N-TTz-Py in chloroform	2.58	2.53	2.64
Bu_2N-TTz-Py in DCM	2.57	2.51	2.62
Bu_2N-TTz-Py in methanol	2.56	2.50	2.62
Ph_2N-TTz-Py in toluene	2.52	2.43	2.55
Ph_2N-TTz-Py in chloroform	2.50	2.41	2.53
Ph_2N-TTz-Py in DCM	2.49	2.40	2.52
Ph_2N-TTz-Py in methanol	2.50	2.40	2.53
Ph_2N-TTz-CHO in toluene	2.36	2.25	2.36
Ph_2N-TTz-CHO in chloroform	2.35	2.22	2.35
Ph_2N-TTz-CHO in DCM	2.35	2.22	2.34
Ph_2N-TTz-CHO in methanol	2.35	2.22	2.34
Ph_2N-TTz-COOH in toluene	2.45	2.33	2.46
Ph_2N-TTz-COOH in chloroform	2.44	2.31	2.45
Ph_2N-TTz-COOH in DCM	2.43	2.30	2.44
Ph_2N-TTz-COOH in methanol	2.44	2.30	2.44
**HandH**			
Bu_2N-TTz-Py in toluene	3.32	3.26	3.37
Bu_2N-TTz-Py in chloroform	3.30	3.23	3.35
Bu_2N-TTz-Py in DCM	3.29	3.22	3.34
Bu_2N-TTz-Py in methanol	3.29	3.22	3.35
Ph_2N-TTz-Py in toluene	3.26	3.19	3.29
Ph_2N-TTz-Py in chloroform	3.25	3.18	3.29
Ph_2N-TTz-Py in DCM	3.25	3.17	3.29
Ph_2N-TTz-Py in methanol	3.26	3.18	3.30

Ph_2N-TTz-CHO in toluene	3.18	3.08	3.19
Ph_2N-TTz-CHO in chloroform	3.17	3.08	3.19
Ph_2N-TTz-CHO in DCM	3.17	3.07	3.18
Ph_2N-TTz-CHO in methanol	3.18	3.08	3.20
Ph_2N-TTz-COOH in toluene	3.22	3.12	3.23
Ph_2N-TTz-COOH in chloroform	3.21	3.12	3.23
Ph_2N-TTz-COOH in DCM	3.21	3.11	3.23
Ph_2N-TTz-COOH in methanol	3.22	3.12	3.24
**Eq. (2)**			
Bu_2N-TTz-Py in toluene	3.02	2.94	3.06
Bu_2N-TTz-Py in chloroform	3.00	2.92	3.04
Bu_2N-TTz-Py in DCM	2.98	2.90	3.03
Bu_2N-TTz-Py in methanol	2.98	2.90	3.04
Ph_2N-TTz-Py in toluene	2.94	2.84	2.96
Ph_2N-TTz-Py in chloroform	2.93	2.83	2.95
Ph_2N-TTz-Py in DCM	2.93	2.82	2.96
Ph_2N-TTz-Py in methanol	2.94	2.83	2.97
Ph_2N-TTz-CHO in toluene	2.82	2.70	2.84
Ph_2N-TTz-CHO in chloroform	2.82	2.69	2.83
Ph_2N-TTz-CHO in DCM	2.82	2.69	2.83
Ph_2N-TTz-CHO in methanol	2.83	2.70	2.85
Ph_2N-TTz-COOH in toluene	2.88	2.76	2.91
Ph_2N-TTz-COOH in chloroform	2.87	2.75	2.90
Ph_2N-TTz-COOH in DCM	2.87	2.74	2.90
Ph_2N-TTz-COOH in methanol	2.88	2.75	2.91

Table S2. Emission spectra changes according to basis sets. Here, the results of Eq. (2) are obtained by using the same dielectric constants described in the manuscript.

	3-21G	6-31G	6-31G*
**B3LYP**			
Bu_2N-TTz-Py in toluene	2.43	2.38	2.46
Bu_2N-TTz-Py in chloroform	2.32	2.28	2.35
Bu_2N-TTz-Py in DCM	2.26	2.22	2.29
Bu_2N-TTz-Py in methanol	2.21	2.16	2.24
Ph_2N-TTz-Py in toluene	1.87	1.84	2.07
Ph_2N-TTz-Py in chloroform	1.93	1.92	2.13
Ph_2N-TTz-Py in DCM	1.99	1.95	2.11
Ph_2N-TTz-Py in methanol	2.00	1.95	2.09
Ph_2N-TTz-CHO in toluene	1.82	1.78	2.02
Ph_2N-TTz-CHO in chloroform	1.93	1.85	2.01
Ph_2N-TTz-CHO in DCM	1.94	1.84	1.98
Ph_2N-TTz-CHO in methanol	1.92	1.82	1.95
Ph_2N-TTz-COOH in toluene	1.88	1.83	2.09
Ph_2N-TTz-COOH in chloroform	1.99	1.90	2.08
Ph_2N-TTz-COOH in DCM	2.00	1.90	2.05
Ph_2N-TTz-COOH in methanol	1.98	1.88	2.02
**HandH**			
Bu_2N-TTz-Py in toluene	2.80	2.77	2.82
Bu_2N-TTz-Py in chloroform	2.68	2.65	2.71
Bu_2N-TTz-Py in DCM	2.61	2.58	2.64
Bu_2N-TTz-Py in methanol	2.55	2.52	2.59
Ph_2N-TTz-Py in toluene	2.74	2.70	2.75
Ph_2N-TTz-Py in chloroform	2.64	2.60	2.66
Ph_2N-TTz-Py in DCM	2.58	2.54	2.60
Ph_2N-TTz-Py in methanol	2.53	2.49	2.55

Ph_2N-TTz-CHO in toluene	2.62	2.57	2.63
Ph_2N-TTz-CHO in chloroform	2.52	2.47	2.53
Ph_2N-TTz-CHO in DCM	2.47	2.42	2.47
Ph_2N-TTz-CHO in methanol	2.42	2.36	2.42
Ph_2N-TTz-COOH in toluene	2.66	2.61	2.67
Ph_2N-TTz-COOH in chloroform	2.56	2.51	2.57
Ph_2N-TTz-COOH in DCM	2.51	2.45	2.52
Ph_2N-TTz-COOH in methanol	2.46	2.40	2.47
**Eq. (2)**			
Bu_2N-TTz-Py in toluene	2.49	2.47	2.53
Bu_2N-TTz-Py in chloroform	2.37	2.36	2.42
Bu_2N-TTz-Py in DCM	2.31	2.29	2.35
Bu_2N-TTz-Py in methanol	2.24	2.23	2.29
Ph_2N-TTz-Py in toluene	2.41	2.38	2.44
Ph_2N-TTz-Py in chloroform	2.31	2.28	2.34
Ph_2N-TTz-Py in DCM	2.25	2.22	2.29
Ph_2N-TTz-Py in methanol	2.20	2.17	2.23
Ph_2N-TTz-CHO in toluene	2.30	2.25	2.32
Ph_2N-TTz-CHO in chloroform	2.20	2.15	2.21
Ph_2N-TTz-CHO in DCM	2.14	2.09	2.16
Ph_2N-TTz-CHO in methanol	2.08	2.04	2.10
Ph_2N-TTz-COOH in toluene	2.33	2.29	2.35
Ph_2N-TTz-COOH in chloroform	2.23	2.19	2.26
Ph_2N-TTz-COOH in DCM	2.17	2.13	2.20
Ph_2N-TTz-COOH in methanol	2.12	2.08	2.15

Table S3. Dipole moment [debye] of asymmetric TTz dyes in the ground and excited states calculated by the dielectric-dependent density functional theory (DFT) method using Eq. (2).

	toluene	chloroform	dichloromethane	methanol
**Ground-state**				
Bu2N-TTz-Py	8.78	9.58	9.85	10.1
Ph2N-TTz-Py	6.23	6.62	6.78	6.93
Ph2N-TTz-CHO	6.88	7.18	7.34	7.51
Ph2N-TTz-COOH	4.86	5.13	5.28	5.44
**Excited-state**				
Bu2N-TTz-Py	18.70	21.12	22.68	24.10
Ph2N-TTz-Py	17.14	18.86	19.91	20.91
Ph2N-TTz-CHO	20.77	22.81	24.04	25.24
Ph2N-TTz-COOH	16.89	18.62	19.66	20.64

Table S4. Charge distributions in the asymmetric TTz dyes in the ground and excited states.

	ground-state			excited-state		
	Acceptor	TTz-core	Donor	Acceptor	TTz-core	Donor
**Bu_2N-TTz-Py**						
toluene	0.009	-0.106	0.096	-0.246	0.032	0.214
chloroform	-0.002	-0.120	0.122	-0.277	0.039	0.239
dichloromethane	0.024	-0.126	0.102	-0.299	0.044	0.255
methanol	0.016	-0.128	0.113	-0.317	0.041	0.276
**Ph_2N-TTz-Py**						
toluene	0.007	-0.102	0.095	-0.230	0.046	0.185
chloroform	0.008	-0.112	0.105	-0.254	0.038	0.216
dichloromethane	0.044	-0.157	0.114	-0.269	0.034	0.235
methanol	0.042	-0.168	0.126	-0.284	0.031	0.252
**Ph_2N-TTz-CHO**						
toluene	-0.005	-0.087	0.092	-0.330	0.143	0.186
chloroform	0.031	-0.134	0.102	-0.357	0.139	0.218
dichloromethane	0.037	-0.144	0.107	-0.375	0.138	0.237
methanol	0.046	-0.161	0.116	-0.393	0.137	0.255
**Ph_2N-TTz-COOH**						
toluene	0.003	-0.086	0.083	-0.301	0.133	0.168
chloroform	0.038	-0.137	0.099	-0.323	0.124	0.199
dichloromethane	0.046	-0.153	0.107	-0.337	0.119	0.218
methanol	0.052	-0.173	0.121	-0.351	0.116	0.236

Table S5. Major contributions (configurational interaction (CI) coefficients) of electronic transition for photon absorption.

	toluene	chloroform	dichloromethane	methanol
**Bu_2N-TTz-Py**				
H → L	0.67	0.67	0.66	0.67
H-1 → L	-0.13	-0.12	-0.12	-0.12
**Ph_2N-TTz-Py**				
H → L	0.66	0.66	0.66	0.66
H-1 → L	0.15	0.15	0.15	0.14
**Ph_2N-TTz-CHO**				
H → L	0.65	0.65	0.65	0.65
H-1 → L	-0.17	-0.17	-0.16	-0.16
**Ph_2N-TTz-COOH**				
H → L	0.65	0.65	0.65	0.65
H-1 → L	0.16	-0.16	-0.16	-0.16

* HOMO=H, HOMO-1=H-1, LUMO=L

Table S6. Major contributions (CI coefficients) of electronic transition for photon emission.

	toluene	chloroform	dichloromethane	methanol
**Bu_2N-TTz-Py**				
$H \rightarrow L$	0.69	0.69	0.69	0.69
**Ph_2N-TTz-Py**				
$H \rightarrow L$	0.69	0.69	0.69	0.69
**Ph_2N-TTz-CHO**				
$H \rightarrow L$	0.69	0.69	0.69	0.69
**Ph_2N-TTz-COOH**				
$H \rightarrow L$	0.69	0.69	0.69	0.69

* HOMO=H, LUMO=L

Table S7. Contributions [eV] of geometrical changes and solvent reorientation effect to the Stokes shift [eV] of asymmetric TTz dyes.

	geometrical change	solvent reorientation	Stokes shift
**Bu_2N-TTz-Py**			
toluene	0.56	0.01	0.57
chloroform	0.57	0.09	0.65
dichloromethane	0.59	0.13	0.72
methanol	0.60	0.19	0.79
**Ph_2N-TTz-Py**			
toluene	0.54	0.01	0.54
chloroform	0.56	0.07	0.64
dichloromethane	0.60	0.11	0.71
methanol	0.64	0.16	0.80
**Ph_2N-TTz-CHO**			
toluene	0.55	0.01	0.56
chloroform	0.60	0.08	0.67
dichloromethane	0.62	0.12	0.74
methanol	0.64	0.17	0.81
**Ph_2N-TTz-COOH**			
toluene	0.59	0.01	0.60
chloroform	0.63	0.07	0.70
dichloromethane	0.65	0.11	0.76
methanol	0.67	0.16	0.84

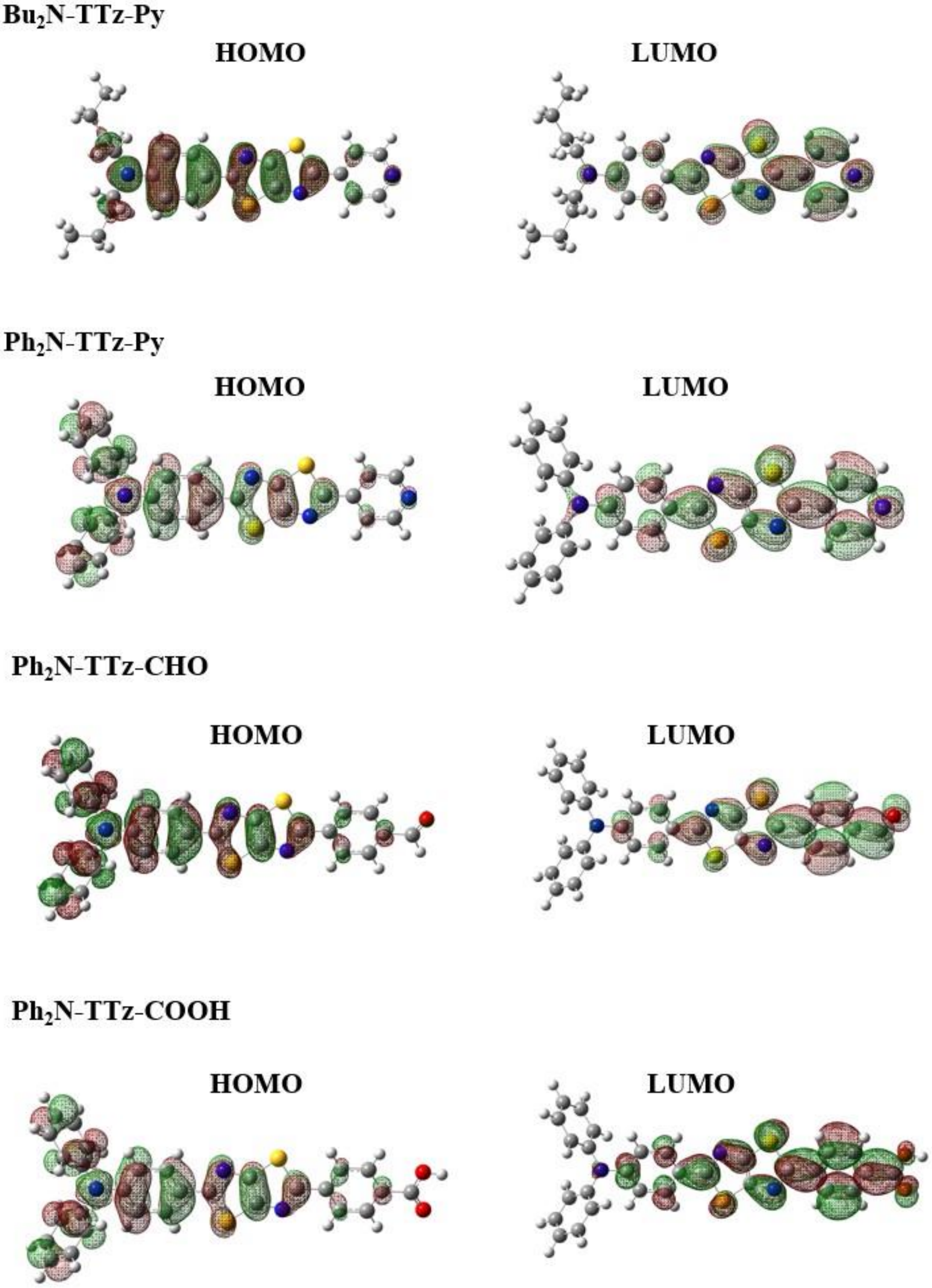

Figure S1. HOMO and LUMO shapes of TTz-based dyes calculated from the dielectric DFT method of Eq. (2). These are obtained by the ground-state optimized structure in DCM.

We show XYZ coordinates of asymmetric TTz dyes for absorption cases. These coordinates are obtained based on the dielectric DFT method of Eq. (2) with the 6-31+G* basis set.

Bu_2N-TTz-Py in absorption state

toluene				chloroform			
**C**	-3.2479	-0.7372	-0.0152	**C**	-3.2400	-0.7477	-0.0094
**C**	-2.8983	0.6194	-0.0760	**C**	-2.8924	0.6047	-0.1442
**S**	-4.3920	1.6034	-0.0586	**S**	-4.3833	1.5904	-0.1431
**C**	-5.3332	0.0238	0.0422	**C**	-5.3244	0.0200	0.0432
**N**	-4.5863	-1.0706	0.0462	**N**	-4.5774	-1.0749	0.0913
**S**	-1.7485	-1.7222	-0.0358	**S**	-1.7399	-1.7323	0.0004
**C**	-0.8057	-0.1302	-0.1526	**C**	-0.7986	-0.1489	-0.2061
**N**	-1.5615	0.9624	-0.1547	**N**	-1.5571	0.9426	-0.2570
**C**	-6.8408	-0.0003	0.0937	**C**	-6.8314	0.0005	0.1174
**C**	0.6909	-0.1159	-0.2131	**C**	0.6969	-0.1316	-0.2809
**C**	-7.5511	-1.2233	-0.1589	**C**	-7.5462	-1.2352	-0.0487
**C**	-8.9750	-1.2123	-0.1007	**C**	-8.9692	-1.2198	0.0305
**N**	-9.7228	-0.1067	0.1776	**N**	-9.7125	-0.0974	0.2515
**C**	-9.0462	1.0491	0.4163	**C**	-9.0320	1.0711	0.4080
**C**	-7.6235	1.1635	0.3920	**C**	-7.6098	1.1816	0.3555
**C**	1.4120	1.1061	0.0366	**C**	1.4116	1.1034	-0.0788
**C**	2.8280	1.1554	-0.0125	**C**	2.8265	1.1583	-0.1282
**C**	3.6243	-0.0208	-0.3315	**C**	3.6335	-0.0230	-0.3977
**C**	2.8876	-1.2511	-0.5623	**C**	2.9028	-1.2665	-0.5888
**C**	1.4675	-1.2861	-0.5087	**C**	1.4828	-1.3070	-0.5343
**N**	5.0298	0.0365	-0.3909	**N**	5.0354	0.0434	-0.4509
**C**	5.7350	1.3337	-0.6488	**C**	5.7476	1.3516	-0.6270
**C**	5.8464	-1.2104	-0.5243	**C**	5.8633	-1.2005	-0.5538
**C**	6.4411	1.9455	0.6240	**C**	6.3543	1.9467	0.7049
**C**	7.2553	3.2680	0.3326	**C**	7.1835	3.2745	0.4893
**C**	6.3721	4.5266	-0.0237	**C**	6.3271	4.5318	0.0696

**C**	5.9484	-2.0599	0.8056	**C**	5.9631	-2.0217	0.7949
**C**	6.6570	-3.4587	0.6151	**C**	6.6583	-3.4307	0.6356
**C**	8.1820	-3.3825	0.2164	**C**	8.1838	-3.3786	0.2360
**H**	-7.0025	-2.1645	-0.3999	**H**	-7.0033	-2.1909	-0.2411
**H**	-9.5481	-2.1536	-0.2952	**H**	-9.5441	-2.1714	-0.0966
**H**	-9.6731	1.9466	0.6481	**H**	-9.6540	1.9826	0.5938
**H**	-7.1525	2.1516	0.6201	**H**	-7.1382	2.1820	0.5180
**H**	0.8465	2.0337	0.2960	**H**	0.8422	2.0386	0.1424
**H**	3.3232	2.1228	0.2373	**H**	3.3170	2.1344	0.0805
**H**	3.4180	-2.2007	-0.8015	**H**	3.4373	-2.2224	-0.7907
**H**	0.9641	-2.2619	-0.7276	**H**	0.9857	-2.2938	-0.7132
**H**	6.5176	1.1495	-1.4496	**H**	6.5898	1.1831	-1.3658
**H**	5.0040	2.0628	-1.0972	**H**	5.0516	2.0927	-1.1137
**H**	5.4537	-1.8569	-1.3741	**H**	5.4787	-1.8658	-1.3922
**H**	6.8782	-0.8869	-0.8373	**H**	6.8937	-0.8754	-0.8700
**H**	5.6639	2.1413	1.4296	**H**	5.5191	2.1342	1.4525
**H**	7.1478	1.1665	1.0499	**H**	7.0323	1.1664	1.1730
**H**	7.8785	3.5092	1.2494	**H**	7.7269	3.5115	1.4568
**H**	7.9972	3.0756	-0.5084	**H**	7.9931	3.0907	-0.2889
**H**	5.6171	4.7392	0.7974	**H**	5.4878	4.7186	0.8117
**H**	7.0178	5.4508	-0.1406	**H**	6.9717	5.4640	0.0388
**H**	5.8001	4.3937	-0.9928	**H**	5.8609	4.4150	-0.9567
**H**	6.5093	-1.4494	1.5824	**H**	6.5315	-1.4004	1.5568
**H**	4.9117	-2.2301	1.2267	**H**	4.9262	-2.1742	1.2228
**H**	6.5621	-4.0368	1.5867	**H**	6.5583	-3.9848	1.6211
**H**	6.0929	-4.0642	-0.1659	**H**	6.0868	-4.0473	-0.1314
**H**	8.7687	-2.7646	0.9674	**H**	8.7761	-2.7491	0.9732
**H**	8.6436	-4.4173	0.1904	**H**	8.6348	-4.4188	0.2342
**H**	8.3380	-2.9209	-0.8070	**H**	8.3438	-2.9412	-0.7976

Bu_2N-TTz-Py in absorption state

DCM				methanol			
**C**	-3.2334	-0.7415	0.0137	**C**	-3.2327	-0.7405	-0.0036
**C**	-2.8923	0.6101	-0.1352	**C**	-2.8950	0.6143	-0.1300
**S**	-4.3909	1.5861	-0.1654	**S**	-4.3957	1.5871	-0.1465
**C**	-5.3225	0.0130	0.0430	**C**	-5.3248	0.0075	0.0130
**N**	-4.5700	-1.0764	0.1082	**N**	-4.5694	-1.0805	0.0742
**S**	-1.7287	-1.7192	0.0427	**S**	-1.7259	-1.7154	0.0047
**C**	-0.7940	-0.1329	-0.1806	**C**	-0.7947	-0.1238	-0.1901
**N**	-1.5573	0.9541	-0.2464	**N**	-1.5605	0.9630	-0.2367
**C**	-6.8297	-0.0133	0.1089	**C**	-6.8313	-0.0214	0.0922
**C**	0.7009	-0.1168	-0.2501	**C**	0.7000	-0.1038	-0.2561
**C**	-7.5403	-1.2413	-0.1182	**C**	-7.5056	-1.1861	0.5955
**C**	-8.9635	-1.2325	-0.0453	**C**	-8.9296	-1.1822	0.6523
**N**	-9.7095	-0.1223	0.2240	**N**	-9.7080	-0.1317	0.2612
**C**	-9.0324	1.0395	0.4377	**C**	-9.0649	0.9712	-0.2135
**C**	-7.6101	1.1547	0.3970	**C**	-7.6462	1.0831	-0.3232
**C**	1.4214	1.1198	-0.0804	**C**	1.4204	1.1240	-0.0298
**C**	2.8372	1.1716	-0.1376	**C**	2.8364	1.1789	-0.0841
**C**	3.6389	-0.0217	-0.3832	**C**	3.6387	-0.0023	-0.3837
**C**	2.9006	-1.2676	-0.5338	**C**	2.9000	-1.2406	-0.5910
**C**	1.4817	-1.3020	-0.4742	**C**	1.4813	-1.2779	-0.5337
**N**	5.0381	0.0301	-0.4595	**N**	5.0369	0.0528	-0.4571
**C**	5.7566	1.3326	-0.6489	**C**	5.7564	1.3631	-0.5848
**C**	5.8547	-1.2222	-0.5634	**C**	5.8540	-1.1937	-0.6200
**C**	6.3409	1.9529	0.6816	**C**	6.3398	1.9192	0.7741
**C**	7.1298	3.3046	0.4616	**C**	7.1347	3.2763	0.6186
**C**	6.2300	4.5440	0.0805	**C**	6.2416	4.5354	0.2898
**C**	6.0021	-2.0182	0.7959	**C**	6.0005	-2.0530	0.7003
**C**	6.7015	-3.4253	0.6342	**C**	6.7011	-3.4505	0.4724
**C**	8.2138	-3.3673	0.1868	**C**	8.2142	-3.3698	0.0314
**H**	-6.9953	-2.1860	-0.3537	**H**	-6.9327	-2.0779	0.9435
**H**	-9.5356	-2.1778	-0.2202	**H**	-9.4733	-2.0787	1.0420
**H**	-9.6567	1.9401	0.6624	**H**	-9.7156	1.8225	-0.5344

**H**	-7.1387	2.1463	0.6066	**H**	-7.2058	2.0201	-0.7440
**H**	0.8575	2.0626	0.1226	**H**	0.8571	2.0567	0.2173
**H**	3.3248	2.1566	0.0443	**H**	3.3234	2.1547	0.1430
**H**	3.4304	-2.2305	-0.7107	**H**	3.4299	-2.1943	-0.8117
**H**	0.9823	-2.2922	-0.6268	**H**	0.9819	-2.2598	-0.7326
**H**	6.6097	1.1482	-1.3698	**H**	6.6096	1.2117	-1.3126
**H**	5.0675	2.0568	-1.1676	**H**	5.0677	2.1112	-1.0690
**H**	5.4296	-1.9009	-1.3685	**H**	5.4296	-1.8334	-1.4565
**H**	6.8730	-0.9125	-0.9281	**H**	6.8723	-0.8666	-0.9683
**H**	5.5004	2.1177	1.4280	**H**	5.4983	2.0534	1.5256
**H**	7.0397	1.1931	1.1510	**H**	7.0352	1.1361	1.2090
**H**	7.6924	3.5452	1.4170	**H**	7.6934	3.4710	1.5867
**H**	7.9232	3.1510	-0.3393	**H**	7.9311	3.1555	-0.1848
**H**	5.4291	4.7206	0.8658	**H**	5.4356	4.6774	1.0769
**H**	6.8549	5.4874	0.0111	**H**	6.8705	5.4786	0.2692
**H**	5.7111	4.4116	-0.9181	**H**	5.7293	4.4514	-0.7174
**H**	6.5914	-1.3822	1.5293	**H**	6.5888	-1.4524	1.4636
**H**	4.9796	-2.1706	1.2575	**H**	4.9776	-2.2290	1.1528
**H**	6.6357	-3.9675	1.6287	**H**	6.6340	-4.0395	1.4399
**H**	6.1120	-4.0553	-0.1074	**H**	6.1133	-4.0441	-0.2996
**H**	8.8237	-2.7255	0.8981	**H**	8.8219	-2.7621	0.7739
**H**	8.6716	-4.4043	0.1827	**H**	8.6727	-4.4053	-0.0213
**H**	8.3388	-2.9412	-0.8559	**H**	8.3407	-2.8939	-0.9893

Ph2N-TTz-Py in absorption state

toluene				chloroform			
**C**	1.2273	-0.1019	-0.0628	**C**	1.2282	-0.1005	-0.0616
**N**	1.9800	0.9599	0.2033	**N**	1.9821	0.9680	0.1758
**C**	3.3190	0.6227	0.1452	**C**	3.3213	0.6279	0.1280
**C**	3.6715	-0.6999	-0.1693	**C**	3.6721	-0.7030	-0.1506
**S**	2.1727	-1.6581	-0.4046	**S**	2.1723	-1.6657	-0.3608
**S**	4.8124	1.5817	0.3778	**S**	4.8153	1.5915	0.3348
**C**	5.7585	0.0405	0.0131	**C**	5.7601	0.0408	0.0124
**N**	5.0108	-1.0240	-0.2449	**N**	5.0114	-1.0299	-0.2174
**C**	7.2675	0.0118	0.0160	**C**	7.2693	0.0110	0.0159
**C**	-0.2749	-0.0872	-0.0602	**C**	-0.2739	-0.0858	-0.0591
**C**	8.0661	1.1758	0.2731	**C**	8.0683	1.1787	0.2554
**C**	9.4892	1.0543	0.2546	**C**	9.4913	1.0567	0.2389
**N**	10.1531	-0.1076	0.0056	**N**	10.1547	-0.1101	0.0078
**C**	9.3909	-1.2124	-0.2385	**C**	9.3921	-1.2189	-0.2193
**C**	7.9649	-1.2181	-0.2471	**C**	7.9662	-1.2234	-0.2280
**C**	-1.0553	-1.1042	-0.7121	**C**	-1.0539	-1.1101	-0.7007
**C**	-2.4784	-1.0652	-0.6972	**C**	-2.4770	-1.0716	-0.6863
**C**	-3.1786	-0.0050	-0.0175	**C**	-3.1788	-0.0046	-0.0177
**C**	-2.3983	1.0162	0.6382	**C**	-2.3985	1.0238	0.6279
**C**	-0.9774	0.9772	0.6117	**C**	-0.9774	0.9849	0.6021
**N**	-4.6137	0.0339	0.0076	**N**	-4.6125	0.0338	0.0068
**C**	-5.3872	-1.1982	0.0911	**C**	-5.3873	-1.1988	0.0822
**C**	-5.3217	1.3060	-0.0570	**C**	-5.3223	1.3060	-0.0499
**C**	-6.3194	1.6279	0.9224	**C**	-6.3073	1.6278	0.9421
**C**	-7.0277	2.8683	0.8461	**C**	-7.0179	2.8678	0.8736
**C**	-6.7368	3.8066	-0.1952	**C**	-6.7416	3.8048	-0.1733
**C**	-5.7359	3.4878	-1.1686	**C**	-5.7532	3.4855	-1.1598
**C**	-5.0381	2.2414	-1.1080	**C**	-5.0532	2.2397	-1.1062
**C**	-5.1234	-2.1499	1.1327	**C**	-5.1370	-2.1486	1.1288
**C**	-5.8854	-3.3568	1.2137	**C**	-5.9009	-3.3552	1.2023
**C**	-6.9307	-3.6191	0.2704	**C**	-6.9345	-3.6183	0.2459
**C**	-7.2014	-2.6643	-0.7611	**C**	-7.1917	-2.6650	-0.7909

**C**	-6.4297	-1.4636	-0.8580	**C**	-6.4179	-1.4647	-0.8797
**H**	7.6104	2.1755	0.4868	**H**	7.6140	2.1817	0.4544
**H**	10.1275	1.9528	0.4530	**H**	10.1289	1.9583	0.4238
**H**	9.9533	-2.1595	-0.4411	**H**	9.9530	-2.1697	-0.4072
**H**	7.4049	-2.1615	-0.4542	**H**	7.4075	-2.1704	-0.4210
**H**	-0.5545	-1.9313	-1.2776	**H**	-0.5533	-1.9428	-1.2580
**H**	-3.0538	-1.8632	-1.2280	**H**	-3.0507	-1.8753	-1.2102
**H**	-2.9133	1.8465	1.1819	**H**	-2.9126	1.8590	1.1646
**H**	-0.3961	1.7722	1.1401	**H**	-0.3982	1.7851	1.1250
**H**	-6.5452	0.9003	1.7429	**H**	-6.5209	0.9018	1.7673
**H**	-7.8055	3.1068	1.6165	**H**	-7.7857	3.1068	1.6539
**H**	-7.2879	4.7802	-0.2492	**H**	-7.2944	4.7777	-0.2216
**H**	-5.5042	4.2093	-1.9940	**H**	-5.5335	4.2058	-1.9895
**H**	-4.2640	1.9918	-1.8777	**H**	-4.2899	1.9893	-1.8864
**H**	-4.3149	-1.9435	1.8796	**H**	-4.3386	-1.9411	1.8861
**H**	-5.6690	-4.0913	2.0319	**H**	-5.6956	-4.0882	2.0245
**H**	-7.5320	-4.5615	0.3408	**H**	-7.5372	-4.5602	0.3102
**H**	-8.0141	-2.8585	-1.5077	**H**	-7.9947	-2.8600	-1.5476
**H**	-6.6410	-0.7229	-1.6705	**H**	-6.6177	-0.7257	-1.6968

Ph2N-TTz-Py in absorption state

DCM				methanol			
**C**	1.2293	-0.1104	-0.0385	**C**	1.2308	-0.1027	-0.0520
**N**	1.9842	0.9652	0.1616	**N**	1.9871	0.9697	0.1614
**C**	3.3237	0.6233	0.1231	**C**	3.3268	0.6263	0.1196
**C**	3.6731	-0.7163	-0.1115	**C**	3.6738	-0.7109	-0.1306
**S**	2.1724	-1.6852	-0.2847	**S**	2.1719	-1.6746	-0.3200
**S**	4.8187	1.5933	0.2908	**S**	4.8239	1.5917	0.2988
**C**	5.7611	0.0318	0.0261	**C**	5.7626	0.0365	-0.0052
**N**	5.0130	-1.0463	-0.1692	**N**	5.0138	-1.0409	-0.2012
**C**	7.2702	0.0083	0.0155	**C**	7.2719	0.0091	0.0010
**C**	-0.2728	-0.0947	-0.0374	**C**	-0.2712	-0.0877	-0.0465
**C**	8.0638	1.1013	0.5000	**C**	8.0652	1.1876	-0.2006
**C**	9.4872	0.9949	0.4496	**C**	9.4887	1.0707	-0.1791
**N**	10.1543	-0.0919	-0.0301	**N**	10.1544	-0.1024	0.0172
**C**	9.3964	-1.1314	-0.4869	**C**	9.3961	-1.2223	0.2044
**C**	7.9703	-1.1403	-0.4927	**C**	7.9699	-1.2303	0.2100
**C**	-1.0530	-1.1333	-0.6557	**C**	-1.0517	-1.1076	-0.6953
**C**	-2.4761	-1.0918	-0.6466	**C**	-2.4748	-1.0684	-0.6818
**C**	-3.1785	-0.0071	-0.0069	**C**	-3.1779	-0.0050	-0.0068
**C**	-2.3976	1.0347	0.6172	**C**	-2.3963	1.0179	0.6480
**C**	-0.9766	0.9923	0.5967	**C**	-0.9752	0.9778	0.6230
**N**	-4.6113	0.0361	0.0110	**N**	-4.6095	0.0351	0.0162
**C**	-5.3918	-1.1933	0.0904	**C**	-5.3898	-1.1977	0.0349
**C**	-5.3180	1.3094	-0.0671	**C**	-5.3201	1.3092	0.0001
**C**	-6.2998	1.6508	0.9215	**C**	-6.2887	1.6062	1.0156
**C**	-7.0085	2.8909	0.8323	**C**	-7.0028	2.8464	0.9870
**C**	-6.7329	3.8081	-0.2325	**C**	-6.7454	3.8070	-0.0437
**C**	-5.7474	3.4691	-1.2157	**C**	-5.7728	3.5119	-1.0541
**C**	-5.0496	2.2230	-1.1410	**C**	-5.0698	2.2663	-1.0397
**C**	-5.1663	-2.1257	1.1579	**C**	-5.1767	-2.1729	1.0660
**C**	-5.9360	-3.3285	1.2360	**C**	-5.9468	-3.3781	1.0851
**C**	-6.9506	-3.6044	0.2625	**C**	-6.9490	-3.6137	0.0881
**C**	-7.1828	-2.6681	-0.7958	**C**	-7.1687	-2.6347	-0.9339

**C**	-6.4029	-1.4716	-0.8883	**C**	-6.3882	-1.4352	-0.9672
**H**	7.6030	2.0267	0.9279	**H**	7.6037	2.1885	-0.3912
**H**	10.1215	1.8377	0.8246	**H**	10.1237	1.9790	-0.3366
**H**	9.9604	-2.0155	-0.8792	**H**	9.9590	-2.1763	0.3671
**H**	7.4150	-2.0236	-0.8903	**H**	7.4140	-2.1834	0.3820
**H**	-0.5527	-1.9801	-1.1913	**H**	-0.5516	-1.9368	-1.2579
**H**	-3.0488	-1.9068	-1.1536	**H**	-3.0465	-1.8680	-1.2135
**H**	-2.9110	1.8831	1.1335	**H**	-2.9086	1.8486	1.1932
**H**	-0.3984	1.8036	1.1035	**H**	-0.3978	1.7732	1.1555
**H**	-6.5120	0.9407	1.7608	**H**	-6.4858	0.8623	1.8288
**H**	-7.7737	3.1456	1.6100	**H**	-7.7572	3.0666	1.7854
**H**	-7.2840	4.7810	-0.2972	**H**	-7.3005	4.7795	-0.0612
**H**	-5.5288	4.1736	-2.0590	**H**	-5.5691	4.2507	-1.8714
**H**	-4.2893	1.9566	-1.9188	**H**	-4.3205	2.0341	-1.8389
**H**	-4.3833	-1.9075	1.9283	**H**	-4.4039	-1.9861	1.8546
**H**	-5.7508	-4.0482	2.0746	**H**	-5.7721	-4.1317	1.8956
**H**	-7.5579	-4.5430	0.3301	**H**	-7.5566	-4.5543	0.1094
**H**	-7.9704	-2.8735	-1.5657	**H**	-7.9461	-2.8086	-1.7216
**H**	-6.5823	-0.7464	-1.7222	**H**	-6.5567	-0.6769	-1.7735

Ph2N-TTz-CHO in absorption state

toluene				chloroform			
**C**	-3.8513	-0.0002	-0.0139	**C**	-3.8518	0.0007	-0.0183
**C**	-3.0633	1.0319	0.6153	**C**	-3.0635	1.0395	0.6005
**C**	-1.6425	0.9844	0.5856	**C**	-1.6425	0.9927	0.5694
**C**	-0.9477	-0.0997	-0.0631	**C**	-0.9472	-0.0962	-0.0711
**C**	-1.7360	-1.1275	-0.6885	**C**	-1.7356	-1.1305	-0.6866
**C**	-3.1591	-1.0800	-0.6704	**C**	-3.1587	-1.0843	-0.6665
**C**	0.5546	-0.1231	-0.0692	**C**	0.5551	-0.1194	-0.0783
**N**	1.3146	0.9374	0.1807	**N**	1.3163	0.9460	0.1482
**C**	2.6518	0.5927	0.1206	**C**	2.6539	0.5989	0.0964
**C**	2.9962	-0.7355	-0.1795	**C**	2.9966	-0.7357	-0.1747
**S**	1.4912	-1.6893	-0.3946	**S**	1.4906	-1.6925	-0.3695
**S**	4.1525	1.5455	0.3375	**S**	4.1557	1.5541	0.2957
**C**	5.0897	-0.0103	0.0021	**C**	5.0909	-0.0093	-0.0036
**N**	4.3335	-1.0717	-0.2487	**N**	4.3342	-1.0752	-0.2338
**C**	6.5979	-0.0404	-0.0091	**C**	6.5995	-0.0396	-0.0114
**C**	7.3816	1.0088	0.5968	**C**	7.3801	0.9914	0.6292
**C**	8.8028	0.9580	0.5705	**C**	8.8015	0.9406	0.6069
**C**	9.4755	-0.1462	-0.0568	**C**	9.4765	-0.1451	-0.0504
**C**	8.6986	-1.1936	-0.6558	**C**	8.7024	-1.1744	-0.6841
**C**	7.2742	-1.1460	-0.6378	**C**	7.2778	-1.1262	-0.6705
**C**	10.9954	-0.2252	-0.0943	**C**	10.9955	-0.2260	-0.0847
**O**	11.7510	0.6091	0.3848	**O**	11.7516	0.5940	0.4219
**N**	-5.2865	0.0473	0.0147	**N**	-5.2859	0.0462	0.0124
**C**	-6.0657	-1.1784	0.1331	**C**	-6.0645	-1.1804	0.1322
**C**	-5.9881	1.3207	-0.0832	**C**	-5.9903	1.3192	-0.0791
**C**	-5.8041	-2.1029	1.1997	**C**	-5.8087	-2.0986	1.2057
**C**	-6.5717	-3.3037	1.3150	**C**	-6.5761	-3.2997	1.3227
**C**	-7.6204	-3.5866	0.3812	**C**	-7.6186	-3.5886	0.3832
**C**	-7.8888	-2.6588	-0.6756	**C**	-7.8810	-2.6668	-0.6807
**C**	-7.1117	-1.4647	-0.8066	**C**	-7.1038	-1.4723	-0.8129
**C**	-6.9845	1.6729	0.8874	**C**	-6.9737	1.6728	0.9042
**C**	-7.6869	2.9145	0.7786	**C**	-7.6791	2.9137	0.8015

**C**	-7.3914	3.8241	-0.2868	**C**	-7.3992	3.8203	-0.2711
**C**	-6.3917	3.4752	-1.2514	**C**	-6.4123	3.4696	-1.2486
**C**	-5.6998	2.2273	-1.1582	**C**	-5.7176	2.2225	-1.1609
**H**	-3.5722	1.8780	1.1406	**H**	-3.5713	1.8893	1.1206
**H**	-1.0551	1.7885	1.0934	**H**	-1.0568	1.8015	1.0717
**H**	-1.2417	-1.9709	-1.2357	**H**	-1.2420	-1.9783	-1.2273
**H**	-3.7407	-1.8874	-1.1804	**H**	-3.7392	-1.8967	-1.1694
**H**	6.8797	1.8671	1.1113	**H**	6.8762	1.8325	1.1689
**H**	9.4050	1.7724	1.0453	**H**	9.4001	1.7407	1.1094
**H**	9.2138	-2.0586	-1.1506	**H**	9.2192	-2.0238	-1.2031
**H**	6.6760	-1.9605	-1.1132	**H**	6.6825	-1.9252	-1.1751
**H**	11.4150	-1.1535	-0.6206	**H**	11.4158	-1.1382	-0.6349
**H**	-4.9929	-1.8800	1.9392	**H**	-5.0033	-1.8707	1.9499
**H**	-6.3571	-4.0168	2.1526	**H**	-6.3665	-4.0078	2.1657
**H**	-8.2262	-4.5240	0.4783	**H**	-8.2240	-4.5260	0.4815
**H**	-8.7040	-2.8694	-1.4152	**H**	-8.6909	-2.8823	-1.4247
**H**	-7.3212	-0.7451	-1.6387	**H**	-7.3079	-0.7582	-1.6511
**H**	-7.2139	0.9680	1.7268	**H**	-7.1902	0.9709	1.7495
**H**	-8.4638	3.1767	1.5426	**H**	-8.4455	3.1774	1.5754
**H**	-7.9379	4.7988	-0.3663	**H**	-7.9479	4.7941	-0.3461
**H**	-6.1564	4.1741	-2.0953	**H**	-6.1897	4.1658	-2.0981
**H**	-4.9266	1.9541	-1.9211	**H**	-4.9556	1.9473	-1.9343

Ph2N-TTz-CHO in absorption state

DCM				methanol			
C	-3.8520	0.0005	-0.0175	C	-3.8520	-0.0004	-0.0120
C	-3.0635	1.0427	0.5959	C	-3.0630	1.0459	0.5947
C	-1.6425	0.9956	0.5654	C	-1.6419	0.9981	0.5648
C	-0.9468	-0.0965	-0.0694	C	-0.9460	-0.0983	-0.0626
C	-1.7351	-1.1345	-0.6792	C	-1.7345	-1.1408	-0.6648
C	-3.1582	-1.0883	-0.6595	C	-3.1577	-1.0942	-0.6456
C	0.5555	-0.1198	-0.0768	C	0.5562	-0.1216	-0.0708
N	1.3173	0.9490	0.1333	N	1.3186	0.9519	0.1133
C	2.6552	0.6005	0.0872	C	2.6567	0.6019	0.0750
C	2.9969	-0.7381	-0.1636	C	2.9974	-0.7423	-0.1442
S	1.4904	-1.6971	-0.3438	S	1.4903	-1.7048	-0.3005
S	4.1575	1.5579	0.2717	S	4.1595	1.5629	0.2353
C	5.0918	-0.0098	-0.0040	C	5.0930	-0.0108	-0.0040
N	4.3347	-1.0790	-0.2179	N	4.3354	-1.0848	-0.1916
C	6.6005	-0.0398	-0.0124	C	6.6018	-0.0403	-0.0137
C	7.3802	0.9900	0.6316	C	7.3810	0.9940	0.6236
C	8.8017	0.9392	0.6096	C	8.8026	0.9433	0.6013
C	9.4772	-0.1446	-0.0507	C	9.4782	-0.1444	-0.0528
C	8.7039	-1.1723	-0.6884	C	8.7053	-1.1763	-0.6846
C	7.2791	-1.1240	-0.6749	C	7.2803	-1.1281	-0.6705
C	10.9957	-0.2262	-0.0851	C	10.9963	-0.2268	-0.0875
O	11.7524	0.5924	0.4255	O	11.7536	0.5946	0.4199
N	-5.2854	0.0463	0.0125	N	-5.2844	0.0468	0.0172
C	-6.0652	-1.1803	0.1284	C	-6.0672	-1.1794	0.1222
C	-5.9901	1.3196	-0.0763	C	-5.9886	1.3210	-0.0705
C	-5.8186	-2.0959	1.2062	C	-5.8366	-2.0945	1.2039
C	-6.5876	-3.2966	1.3198	C	-6.6087	-3.2943	1.3075
C	-7.6224	-3.5873	0.3721	C	-7.6307	-3.5841	0.3452
C	-7.8757	-2.6678	-0.6964	C	-7.8680	-2.6648	-0.7274
C	-7.0968	-1.4738	-0.8248	C	-7.0857	-1.4716	-0.8452
C	-6.9656	1.6756	0.9138	C	-6.9559	1.6806	0.9264
C	-7.6717	2.9166	0.8138	C	-7.6623	2.9218	0.8279

**C**	-7.4000	3.8205	-0.2635	**C**	-7.3986	3.8219	-0.2548
**C**	-6.4207	3.4671	-1.2482	**C**	-6.4274	3.4647	-1.2464
**C**	-5.7256	2.2200	-1.1626	**C**	-5.7321	2.2173	-1.1620
**H**	-3.5707	1.8950	1.1125	**H**	-3.5694	1.9013	1.1066
**H**	-1.0578	1.8068	1.0652	**H**	-1.0581	1.8124	1.0608
**H**	-1.2417	-1.9852	-1.2154	**H**	-1.2413	-1.9955	-1.1948
**H**	-3.7379	-1.9034	-1.1588	**H**	-3.7366	-1.9128	-1.1400
**H**	6.8760	1.8293	1.1737	**H**	6.8768	1.8364	1.1607
**H**	9.3988	1.7385	1.1152	**H**	9.3986	1.7462	1.1023
**H**	9.2208	-2.0195	-1.2103	**H**	9.2220	-2.0258	-1.2024
**H**	6.6850	-1.9213	-1.1838	**H**	6.6871	-1.9281	-1.1761
**H**	11.4162	-1.1355	-0.6380	**H**	11.4167	-1.1377	-0.6361
**H**	-5.0198	-1.8663	1.9570	**H**	-5.0484	-1.8651	1.9659
**H**	-6.3855	-4.0027	2.1662	**H**	-6.4195	-4.0002	2.1570
**H**	-8.2290	-4.5242	0.4675	**H**	-8.2398	-4.5202	0.4326
**H**	-8.6792	-2.8849	-1.4467	**H**	-8.6610	-2.8815	-1.4889
**H**	-7.2929	-0.7621	-1.6669	**H**	-7.2684	-0.7608	-1.6910
**H**	-7.1750	0.9763	1.7631	**H**	-7.1580	0.9847	1.7803
**H**	-8.4318	3.1825	1.5930	**H**	-8.4157	3.1910	1.6125
**H**	-7.9491	4.7942	-0.3367	**H**	-7.9477	4.7956	-0.3270
**H**	-6.2050	4.1610	-2.1013	**H**	-6.2185	4.1554	-2.1037
**H**	-4.9703	1.9424	-1.9418	**H**	-4.9840	1.9362	-1.9467

Ph2N-TTz-COOH in absorption state

toluene				chloroform			
C	0.1992	-0.0931	-0.0684	C	0.2000	-0.0971	-0.0531
N	0.9508	0.9610	0.2295	N	0.9529	0.9761	0.1644
C	2.2913	0.6278	0.1602	C	2.2937	0.6374	0.1213
C	2.6455	-0.6838	-0.1952	C	2.6462	-0.6975	-0.1336
S	1.1476	-1.6364	-0.4608	S	1.1473	-1.6669	-0.3230
S	3.7856	1.5821	0.4185	S	3.7891	1.6060	0.3114
C	4.7342	0.0558	-0.0119	C	4.7357	0.0449	0.0329
N	3.9863	-1.0034	-0.2925	N	3.9873	-1.0287	-0.1863
C	6.2434	0.0341	-0.0048	C	6.2453	0.0264	0.0276
C	-1.3041	-0.0814	-0.0628	C	-1.3033	-0.0841	-0.0525
C	7.0185	1.2436	-0.0753	C	7.0172	1.0570	0.6688
C	8.4442	1.1948	-0.0633	C	8.4432	1.0163	0.6461
C	8.3529	-1.2785	0.0772	C	8.3577	-1.0946	-0.6514
C	6.9306	-1.2319	0.0708	C	6.9351	-1.0538	-0.6343
C	-2.0834	-1.0771	-0.7489	C	-2.0817	-1.1269	-0.6666
C	-3.5071	-1.0410	-0.7315	C	-3.5055	-1.0897	-0.6544
C	-4.2084	-0.0060	-0.0149	C	-4.2090	-0.0056	-0.0158
C	-3.4296	0.9934	0.6753	C	-3.4307	1.0414	0.6021
C	-2.0079	0.9581	0.6463	C	-2.0090	1.0037	0.5788
N	-5.6450	0.0296	0.0126	N	-5.6442	0.0314	0.0067
C	-6.4151	-1.2063	0.0684	C	-6.4156	-1.1995	0.1316
C	-6.3547	1.3019	-0.0244	C	-6.3558	1.2990	-0.1044
C	-7.3596	1.5978	0.9566	C	-7.3515	1.6558	0.8657
C	-8.0693	2.8393	0.9072	C	-8.0640	2.8912	0.7439
C	-7.7729	3.8048	-0.1080	C	-7.7790	3.7897	-0.3346
C	-6.7647	3.5119	-1.0828	C	-6.7798	3.4359	-1.2989
C	-6.0655	2.2648	-1.0495	C	-6.0780	2.1940	-1.1923
C	-6.1424	-2.1850	1.0832	C	-6.1603	-2.1073	1.2144
C	-6.9005	-3.3965	1.1364	C	-6.9200	-3.3131	1.3364
C	-7.9511	-3.6371	0.1925	C	-7.9546	-3.6171	0.3926
C	-8.2308	-2.6556	-0.8119	C	-8.2168	-2.7056	-0.6806
C	-7.4630	-1.4501	-0.8816	C	-7.4471	-1.5066	-0.8177

**H**	6.5144	2.2398	-0.1597	**H**	6.5112	1.8944	1.2128
**H**	9.0278	2.1455	-0.1223	**H**	9.0235	1.8243	1.1540
**H**	8.8806	-2.2623	0.1392	**H**	8.8856	-1.9327	-1.1700
**H**	6.3388	-2.1776	0.1303	**H**	6.3466	-1.8567	-1.1415
**H**	-1.5812	-1.8837	-1.3431	**H**	-1.5794	-1.9744	-1.2003
**H**	-4.0822	-1.8216	-1.2890	**H**	-4.0783	-1.9087	-1.1562
**H**	-3.9462	1.8038	1.2479	**H**	-3.9471	1.8909	1.1151
**H**	-1.4273	1.7357	1.2017	**H**	-1.4309	1.8191	1.0801
**H**	-7.5900	0.8491	1.7572	**H**	-7.5722	0.9605	1.7157
**H**	-8.8529	3.0571	1.6788	**H**	-8.8401	3.1574	1.5076
**H**	-8.3251	4.7793	-0.1407	**H**	-8.3333	4.7593	-0.4245
**H**	-6.5283	4.2549	-1.8882	**H**	-6.5530	4.1256	-2.1529
**H**	-5.2857	2.0361	-1.8207	**H**	-5.3063	1.9165	-1.9555
**H**	-5.3297	-1.9961	1.8308	**H**	-5.3608	-1.8677	1.9619
**H**	-6.6767	-4.1520	1.9338	**H**	-6.7106	-4.0130	2.1866
**H**	-8.5494	-4.5833	0.2413	**H**	-8.5542	-4.5582	0.4947
**H**	-9.0476	-2.8325	-1.5591	**H**	-9.0206	-2.9331	-1.4281
**H**	-7.6813	-0.6886	-1.6735	**H**	-7.6509	-0.8008	-1.6633
**C**	9.1230	-0.0673	0.0129	**C**	9.1250	-0.0604	-0.0143
**C**	10.6506	-0.1713	0.0267	**C**	10.6532	-0.1474	-0.0631
**O**	11.2714	-1.2225	0.0858	**O**	11.2763	-1.0461	-0.6120
**O**	11.2667	1.0435	-0.0342	**O**	11.2649	0.8887	0.5733
**H**	12.2419	0.8897	-0.0207	**H**	12.2418	0.7631	0.4985

Ph2N-TTz-COOH in absorption state

DCM				methanol			
C	0.2005	-0.0984	-0.0491	C	0.2015	-0.1010	-0.0410
N	0.9540	0.9788	0.1471	N	0.9557	0.9823	0.1175
C	2.2951	0.6385	0.1114	C	2.2970	0.6402	0.0941
C	2.6465	-0.7013	-0.1169	C	2.6473	-0.7069	-0.0873
S	1.1470	-1.6735	-0.2873	S	1.1470	-1.6835	-0.2236
S	3.7910	1.6099	0.2819	S	3.7934	1.6164	0.2289
C	4.7367	0.0438	0.0341	C	4.7380	0.0428	0.0354
N	3.9878	-1.0340	-0.1635	N	3.9889	-1.0415	-0.1234
C	6.2464	0.0256	0.0277	C	6.2479	0.0248	0.0274
C	-1.3027	-0.0853	-0.0487	C	-1.3016	-0.0880	-0.0402
C	7.0180	1.0592	0.6645	C	7.0195	1.0714	0.6427
C	8.4442	1.0188	0.6410	C	8.4458	1.0310	0.6182
C	8.3588	-1.0974	-0.6484	C	8.3600	-1.1103	-0.6296
C	6.9361	-1.0569	-0.6306	C	6.9372	-1.0700	-0.6108
C	-2.0810	-1.1325	-0.6559	C	-2.0797	-1.1423	-0.6355
C	-3.5048	-1.0950	-0.6449	C	-3.5036	-1.1041	-0.6264
C	-4.2091	-0.0060	-0.0145	C	-4.2089	-0.0073	-0.0096
C	-3.4307	1.0450	0.5971	C	-3.4301	1.0502	0.5915
C	-2.0089	1.0067	0.5751	C	-2.0083	1.0109	0.5711
N	-5.6435	0.0317	0.0065	N	-5.6421	0.0322	0.0088
C	-6.4170	-1.1991	0.1251	C	-6.4195	-1.1981	0.1130
C	-6.3551	1.3001	-0.1009	C	-6.3531	1.3021	-0.0926
C	-7.3424	1.6591	0.8768	C	-7.3291	1.6630	0.8957
C	-8.0553	2.8949	0.7588	C	-8.0423	2.8995	0.7839
C	-7.7787	3.7911	-0.3241	C	-7.7769	3.7936	-0.3037
C	-6.7876	3.4349	-1.2963	C	-6.7971	3.4351	-1.2867
C	-6.0856	2.1926	-1.1929	C	-6.0949	2.1923	-1.1890
C	-6.1735	-2.1049	1.2122	C	-6.1973	-2.1041	1.2043
C	-6.9356	-3.3100	1.3284	C	-6.9640	-3.3077	1.3070
C	-7.9607	-3.6148	0.3741	C	-7.9723	-3.6104	0.3339
C	-8.2109	-2.7049	-0.7037	C	-8.2012	-2.7002	-0.7486
C	-7.4386	-1.5066	-0.8346	C	-7.4241	-1.5032	-0.8652

**H**	6.5123	1.8986	1.2054	**H**	6.5142	1.9212	1.1674
**H**	9.0237	1.8292	1.1456	**H**	9.0249	1.8518	1.1061
**H**	8.8856	-1.9374	-1.1649	**H**	8.8855	-1.9601	-1.1309
**H**	6.3484	-1.8615	-1.1360	**H**	6.3500	-1.8842	-1.1013
**H**	-1.5788	-1.9838	-1.1835	**H**	-1.5773	-1.9996	-1.1529
**H**	-4.0767	-1.9172	-1.1423	**H**	-4.0742	-1.9316	-1.1162
**H**	-3.9465	1.8976	1.1052	**H**	-3.9450	1.9081	1.0912
**H**	-1.4320	1.8251	1.0730	**H**	-1.4324	1.8343	1.0621
**H**	-7.5557	0.9660	1.7306	**H**	-7.5326	0.9718	1.7534
**H**	-8.8247	3.1631	1.5285	**H**	-8.8026	3.1698	1.5618
**H**	-8.3331	4.7608	-0.4114	**H**	-8.3314	4.7638	-0.3863
**H**	-6.5679	4.1226	-2.1537	**H**	-6.5866	4.1212	-2.1477
**H**	-5.3211	1.9128	-1.9625	**H**	-5.3400	1.9100	-1.9671
**H**	-5.3823	-1.8643	1.9679	**H**	-5.4198	-1.8645	1.9746
**H**	-6.7359	-4.0085	2.1820	**H**	-6.7815	-4.0066	2.1641
**H**	-8.5620	-4.5552	0.4715	**H**	-8.5772	-4.5495	0.4204
**H**	-9.0068	-2.9332	-1.4593	**H**	-8.9834	-2.9270	-1.5187
**H**	-7.6324	-0.8025	-1.6840	**H**	-7.6000	-0.7993	-1.7187
**C**	9.1261	-0.0604	-0.0155	**C**	9.1275	-0.0607	-0.0179
**C**	10.6544	-0.1460	-0.0648	**C**	10.6560	-0.1455	-0.0673
**O**	11.2775	-1.0470	-0.6120	**O**	11.2788	-1.0568	-0.5992
**O**	11.2652	0.8915	0.5674	**O**	11.2662	0.9029	0.5443
**H**	12.2427	0.7691	0.4939	**H**	12.2443	0.7824	0.4730

We show XYZ coordinates of asymmetric TTz dyes for emission cases. These coordinates are obtained based on the dielectric DFT method of Eq. (2) with the 6-31+G* basis set.

Bu_2N-TTz-Py in emission

**toluene**				**chloroform**			
**C**	-3.2332	-0.7783	-0.1413	**C**	-3.2312	-0.7802	-0.1378
**C**	-2.8478	0.6196	-0.0045	**C**	-2.8459	0.6209	-0.0230
**S**	-4.3393	1.6145	0.1661	**S**	-4.3388	1.6167	0.1418
**C**	-5.3056	0.0121	0.0499	**C**	-5.3056	0.0131	0.0463
**N**	-4.5364	-1.1023	-0.1123	**N**	-4.5322	-1.1046	-0.1010
**S**	-1.7533	-1.7701	-0.3224	**S**	-1.7493	-1.7725	-0.3087
**C**	-0.7738	-0.1742	-0.2155	**C**	-0.7695	-0.1754	-0.2185
**N**	-1.5492	0.9378	-0.0457	**N**	-1.5494	0.9394	-0.0666
**C**	-6.7663	-0.0080	0.1145	**C**	-6.7628	-0.0090	0.1173
**C**	0.6764	-0.1542	-0.2673	**C**	0.6781	-0.1529	-0.2778
**C**	-7.4993	-1.2625	0.0200	**C**	-7.4972	-1.2663	0.0425
**C**	-8.9167	-1.2373	0.0881	**C**	-8.9137	-1.2425	0.1158
**N**	-9.6711	-0.1031	0.2381	**N**	-9.6707	-0.1069	0.2541
**C**	-8.9862	1.0749	0.3239	**C**	-8.9854	1.0739	0.3230
**C**	-7.5693	1.1923	0.2702	**C**	-7.5696	1.1924	0.2626
**C**	1.4055	1.1092	-0.1449	**C**	1.4075	1.1137	-0.1753
**C**	2.8078	1.1628	-0.2137	**C**	2.8091	1.1681	-0.2451
**C**	3.6217	-0.0445	-0.4125	**C**	3.6254	-0.0424	-0.4260
**C**	2.8899	-1.3108	-0.4969	**C**	2.8924	-1.3115	-0.4977
**C**	1.4829	-1.3592	-0.4297	**C**	1.4867	-1.3596	-0.4303
**N**	5.0087	0.0136	-0.4969	**N**	5.0087	0.0138	-0.5103
**C**	5.7282	1.3173	-0.6964	**C**	5.7339	1.3191	-0.6917
**C**	5.8433	-1.2346	-0.5288	**C**	5.8436	-1.2368	-0.5367
**C**	6.2820	1.9672	0.6346	**C**	6.2602	1.9640	0.6530
**C**	7.0440	3.3319	0.3967	**C**	7.0278	3.3290	0.4347
**C**	6.1197	4.5474	-0.0014	**C**	6.1131	4.5476	0.0241
**C**	5.9917	-1.9555	0.8734	**C**	5.9937	-1.9497	0.8696
**C**	6.7180	-3.3558	0.7831	**C**	6.7175	-3.3516	0.7836

**C**	8.2340	-3.2913	0.3505	**C**	8.2331	-3.2911	0.3488
**H**	-6.9572	-2.2301	-0.1024	**H**	-6.9567	-2.2360	-0.0697
**H**	-9.4912	-2.1959	0.0178	**H**	-9.4857	-2.2034	0.0600
**H**	-9.6121	1.9956	0.4444	**H**	-9.6109	1.9959	0.4347
**H**	-7.1060	2.2073	0.3498	**H**	-7.1089	2.2094	0.3281
**H**	0.8295	2.0515	0.0134	**H**	0.8327	2.0588	-0.0299
**H**	3.2944	2.1556	-0.0805	**H**	3.2934	2.1635	-0.1282
**H**	3.4328	-2.2742	-0.6233	**H**	3.4353	-2.2757	-0.6151
**H**	0.9893	-2.3592	-0.5151	**H**	0.9946	-2.3610	-0.5051
**H**	6.5945	1.1144	-1.3949	**H**	6.6136	1.1151	-1.3712
**H**	5.0473	2.0244	-1.2459	**H**	5.0651	2.0274	-1.2541
**H**	5.4231	-1.9551	-1.2975	**H**	5.4197	-1.9595	-1.2998
**H**	6.8574	-0.9316	-0.9089	**H**	6.8562	-0.9359	-0.9209
**H**	5.4278	2.1260	1.3656	**H**	5.3919	2.1219	1.3672
**H**	6.9899	1.2311	1.1262	**H**	6.9588	1.2268	1.1550
**H**	7.5985	3.5945	1.3504	**H**	7.5635	3.5865	1.4004
**H**	7.8433	3.1841	-0.3991	**H**	7.8419	3.1831	-0.3461
**H**	5.3215	4.7258	0.7857	**H**	5.2986	4.7217	0.7953
**H**	6.7275	5.4995	-0.0919	**H**	6.7241	5.4994	-0.0481
**H**	5.5976	4.3901	-0.9949	**H**	5.6117	4.3957	-0.9808
**H**	6.5633	-1.2743	1.5783	**H**	6.5680	-1.2668	1.5701
**H**	4.9677	-2.1012	1.3326	**H**	4.9704	-2.0929	1.3312
**H**	6.6487	-3.8506	1.8012	**H**	6.6491	-3.8416	1.8041
**H**	6.1481	-4.0309	0.0667	**H**	6.1453	-4.0280	0.0704
**H**	8.8256	-2.6088	1.0388	**H**	8.8261	-2.6069	1.0341
**H**	8.7085	-4.3191	0.3986	**H**	8.7054	-4.3198	0.4000
**H**	8.3660	-2.9121	-0.7094	**H**	8.3638	-2.9153	-0.7125

Bu_2N-TTz-Py in emission state

DCM				methanol			
C	-3.2329	-0.7792	-0.1332	C	-3.2341	-0.7794	-0.1266
C	-2.8474	0.6231	-0.0220	C	-2.8484	0.6243	-0.0209
S	-4.3413	1.6190	0.1401	S	-4.3431	1.6208	0.1360
C	-5.3086	0.0156	0.0461	C	-5.3111	0.0175	0.0460
N	-4.5328	-1.1033	-0.0971	N	-4.5330	-1.1029	-0.0912
S	-1.7501	-1.7713	-0.3001	S	-1.7504	-1.7718	-0.2877
C	-0.7697	-0.1742	-0.2110	C	-0.7695	-0.1743	-0.2032
N	-1.5523	0.9417	-0.0637	N	-1.5545	0.9429	-0.0619
C	-6.7641	-0.0076	0.1153	C	-6.7650	-0.0066	0.1127
C	0.6762	-0.1506	-0.2723	C	0.6747	-0.1497	-0.2659
C	-7.4994	-1.2658	0.0410	C	-7.5011	-1.2658	0.0410
C	-8.9157	-1.2429	0.1120	C	-8.9172	-1.2436	0.1093
N	-9.6744	-0.1072	0.2478	N	-9.6776	-0.1076	0.2404
C	-8.9888	1.0746	0.3168	C	-8.9918	1.0754	0.3072
C	-7.5735	1.1937	0.2585	C	-7.5767	1.1949	0.2511
C	1.4065	1.1171	-0.1748	C	1.4053	1.1195	-0.1756
C	2.8073	1.1716	-0.2468	C	2.8055	1.1746	-0.2497
C	3.6242	-0.0401	-0.4251	C	3.6234	-0.0385	-0.4235
C	2.8902	-1.3105	-0.4926	C	2.8889	-1.3106	-0.4838
C	1.4855	-1.3583	-0.4236	C	1.4851	-1.3583	-0.4130
N	5.0051	0.0150	-0.5125	N	5.0018	0.0161	-0.5146
C	5.7332	1.3205	-0.6898	C	5.7320	1.3224	-0.6872
C	5.8401	-1.2370	-0.5410	C	5.8376	-1.2370	-0.5464
C	6.2613	1.9580	0.6576	C	6.2572	1.9548	0.6635
C	7.0435	3.3151	0.4433	C	7.0515	3.3056	0.4539
C	6.1453	4.5404	0.0169	C	6.1682	4.5354	0.0102
C	5.9929	-1.9493	0.8652	C	5.9964	-1.9478	0.8599
C	6.7144	-3.3523	0.7773	C	6.7163	-3.3514	0.7694
C	8.2285	-3.2938	0.3371	C	8.2274	-3.2945	0.3190
H	-6.9596	-2.2361	-0.0695	H	-6.9621	-2.2370	-0.0658
H	-9.4862	-2.2047	0.0562	H	-9.4864	-2.2063	0.0550
H	-9.6143	1.9968	0.4268	H	-9.6172	1.9979	0.4136

**H**	-7.1142	2.2112	0.3241	**H**	-7.1189	2.2131	0.3146
**H**	0.8328	2.0632	-0.0314	**H**	0.8325	2.0666	-0.0360
**H**	3.2914	2.1676	-0.1340	**H**	3.2886	2.1714	-0.1430
**H**	3.4327	-2.2749	-0.6087	**H**	3.4313	-2.2753	-0.5960
**H**	0.9937	-2.3599	-0.4954	**H**	0.9941	-2.3605	-0.4795
**H**	6.6114	1.1161	-1.3703	**H**	6.6110	1.1176	-1.3658
**H**	5.0654	2.0321	-1.2486	**H**	5.0663	2.0359	-1.2457
**H**	5.4132	-1.9586	-1.3029	**H**	5.4067	-1.9578	-1.3060
**H**	6.8513	-0.9361	-0.9277	**H**	6.8465	-0.9354	-0.9375
**H**	5.3922	2.1242	1.3690	**H**	5.3855	2.1282	1.3699
**H**	6.9516	1.2140	1.1607	**H**	6.9394	1.2058	1.1697
**H**	7.5695	3.5709	1.4149	**H**	7.5658	3.5612	1.4318
**H**	7.8654	3.1584	-0.3269	**H**	7.8823	3.1394	-0.3045
**H**	5.3191	4.7210	0.7741	**H**	5.3289	4.7212	0.7517
**H**	6.7653	5.4873	-0.0454	**H**	6.7950	5.4786	-0.0417
**H**	5.6600	4.3913	-0.9961	**H**	5.7007	4.3874	-1.0113
**H**	6.5707	-1.2675	1.5638	**H**	6.5789	-1.2663	1.5545
**H**	4.9708	-2.0916	1.3299	**H**	4.9766	-2.0895	1.3298
**H**	6.6492	-3.8410	1.7987	**H**	6.6578	-3.8375	1.7926
**H**	6.1385	-4.0280	0.0667	**H**	6.1348	-4.0279	0.0642
**H**	8.8243	-2.6092	1.0195	**H**	8.8275	-2.6073	0.9949
**H**	8.6997	-4.3232	0.3879	**H**	8.6986	-4.3241	0.3703
**H**	8.3555	-2.9192	-0.7250	**H**	8.3469	-2.9235	-0.7451

Ph2N-TTz-Py in emission case

toluene				chloroform			
**C**	1.1939	-0.1222	-0.0817	**C**	1.1938	-0.1223	-0.0843
**N**	1.9773	0.8486	0.4833	**N**	1.9806	0.8645	0.4548
**C**	3.2762	0.5681	0.3256	**C**	3.2774	0.5793	0.3059
**C**	3.6559	-0.6466	-0.3773	**C**	3.6582	-0.6562	-0.3634
**S**	2.1694	-1.5112	-0.8842	**S**	2.1708	-1.5336	-0.8472
**S**	4.7763	1.4269	0.8339	**S**	4.7781	1.4514	0.7918
**C**	5.7372	0.0358	0.0301	**C**	5.7403	0.0383	0.0286
**N**	4.9632	-0.9279	-0.5323	**N**	4.9637	-0.9417	-0.5089
**C**	7.2061	0.0139	0.0234	**C**	7.2065	0.0141	0.0244
**C**	-0.2562	-0.1022	-0.0731	**C**	-0.2537	-0.1011	-0.0759
**C**	8.0156	1.0510	0.6341	**C**	8.0181	1.0670	0.6086
**C**	9.4350	0.9453	0.5785	**C**	9.4368	0.9585	0.5586
**N**	10.1130	-0.0787	-0.0182	**N**	10.1160	-0.0827	-0.0095
**C**	9.3508	-1.0598	-0.5956	**C**	9.3527	-1.0789	-0.5621
**C**	7.9299	-1.0760	-0.6113	**C**	7.9324	-1.0937	-0.5798
**C**	-1.0649	-1.1562	-0.6894	**C**	-1.0641	-1.1703	-0.6664
**C**	-2.4754	-1.1095	-0.6615	**C**	-2.4734	-1.1242	-0.6375
**C**	-3.1728	-0.0100	-0.0196	**C**	-3.1729	-0.0089	-0.0212
**C**	-2.3813	1.0458	0.5963	**C**	-2.3795	1.0633	0.5668
**C**	-0.9741	1.0037	0.5742	**C**	-0.9732	1.0208	0.5444
**N**	-4.5957	0.0329	0.0124	**N**	-4.5916	0.0330	0.0116
**C**	-5.3629	-1.1880	0.1848	**C**	-5.3615	-1.1891	0.1782
**C**	-5.2963	1.2964	-0.1317	**C**	-5.2964	1.2972	-0.1246
**C**	-6.3618	1.6392	0.7715	**C**	-6.3451	1.6413	0.7964
**C**	-7.0504	2.8804	0.6207	**C**	-7.0389	2.8811	0.6522
**C**	-6.6833	3.7936	-0.4211	**C**	-6.6926	3.7892	-0.4013
**C**	-5.6192	3.4520	-1.3172	**C**	-5.6453	3.4446	-1.3164
**C**	-4.9281	2.2101	-1.1797	**C**	-4.9496	2.2042	-1.1850
**C**	-5.0124	-2.1187	1.2239	**C**	-5.0269	-2.1127	1.2281
**C**	-5.7698	-3.3175	1.3910	**C**	-5.7873	-3.3106	1.3909
**C**	-6.8827	-3.5985	0.5335	**C**	-6.8872	-3.5959	0.5177
**C**	-7.2315	-2.6685	-0.4994	**C**	-7.2205	-2.6715	-0.5256

**C**	-6.4768	-1.4700	-0.6796	**C**	-6.4624	-1.4738	-0.7009
**H**	7.5575	1.9334	1.1487	**H**	7.5613	1.9637	1.0988
**H**	10.0675	1.7424	1.0479	**H**	10.0682	1.7679	1.0079
**H**	9.9197	-1.8940	-1.0817	**H**	9.9204	-1.9270	-1.0248
**H**	7.3804	-1.9136	-1.1048	**H**	7.3851	-1.9449	-1.0519
**H**	-0.5714	-2.0124	-1.2144	**H**	-0.5718	-2.0381	-1.1729
**H**	-3.0618	-1.9222	-1.1569	**H**	-3.0586	-1.9485	-1.1140
**H**	-2.8980	1.8921	1.1130	**H**	-2.8946	1.9218	1.0640
**H**	-0.3866	1.8117	1.0724	**H**	-0.3876	1.8416	1.0235
**H**	-6.6439	0.9338	1.5929	**H**	-6.6107	0.9395	1.6263
**H**	-7.8747	3.1421	1.3323	**H**	-7.8506	3.1449	1.3772
**H**	-7.2249	4.7671	-0.5338	**H**	-7.2381	4.7610	-0.5093
**H**	-5.3311	4.1535	-2.1414	**H**	-5.3748	4.1415	-2.1501
**H**	-4.1080	1.9389	-1.8910	**H**	-4.1440	1.9294	-1.9114
**H**	-4.1540	-1.8936	1.9055	**H**	-4.1799	-1.8831	1.9225
**H**	-5.4953	-4.0321	2.2085	**H**	-5.5260	-4.0202	2.2169
**H**	-7.4762	-4.5380	0.6698	**H**	-7.4832	-4.5343	0.6505
**H**	-8.0940	-2.8832	-1.1809	**H**	-8.0732	-2.8893	-1.2180
**H**	-6.7457	-0.7510	-1.4937	**H**	-6.7191	-0.7591	-1.5226

Ph2N-TTz-Py in emission case

DCM				methanol			
C	1.1938	-0.1231	-0.0840	C	1.1937	-0.1257	-0.0810
N	1.9827	0.8745	0.4363	N	1.9845	0.8812	0.4226
C	3.2782	0.5862	0.2934	C	3.2788	0.5909	0.2843
C	3.6594	-0.6634	-0.3517	C	3.6605	-0.6707	-0.3396
S	2.1715	-1.5496	-0.8187	S	2.1722	-1.5649	-0.7906
S	4.7792	1.4680	0.7619	S	4.7801	1.4813	0.7369
C	5.7421	0.0396	0.0278	C	5.7437	0.0405	0.0277
N	4.9637	-0.9516	-0.4909	N	4.9637	-0.9607	-0.4740
C	7.2065	0.0143	0.0245	C	7.2064	0.0147	0.0238
C	-0.2522	-0.1012	-0.0755	C	-0.2508	-0.1029	-0.0726
C	8.0198	1.0793	0.5864	C	8.0215	1.0901	0.5656
C	9.4382	0.9690	0.5392	C	9.4395	0.9790	0.5193
N	10.1177	-0.0851	-0.0062	N	10.1193	-0.0855	-0.0076
C	9.3534	-1.0929	-0.5380	C	9.3539	-1.1032	-0.5203
C	7.9333	-1.1071	-0.5557	C	7.9341	-1.1175	-0.5366
C	-1.0636	-1.1794	-0.6498	C	-1.0632	-1.1888	-0.6326
C	-2.4721	-1.1335	-0.6209	C	-2.4710	-1.1425	-0.6042
C	-3.1728	-0.0085	-0.0208	C	-3.1727	-0.0089	-0.0192
C	-2.3784	1.0730	0.5505	C	-2.3772	1.0805	0.5377
C	-0.9726	1.0303	0.5280	C	-0.9719	1.0370	0.5159
N	-4.5890	0.0333	0.0115	N	-4.5865	0.0337	0.0116
C	-5.3612	-1.1895	0.1727	C	-5.3617	-1.1891	0.1672
C	-5.2958	1.2982	-0.1198	C	-5.2942	1.2997	-0.1160
C	-6.3349	1.6427	0.8113	C	-6.3233	1.6466	0.8247
C	-7.0313	2.8819	0.6716	C	-7.0213	2.8856	0.6882
C	-6.6967	3.7877	-0.3879	C	-6.6977	3.7877	-0.3780
C	-5.6590	3.4416	-1.3136	C	-5.6700	3.4382	-1.3138
C	-4.9610	2.2018	-1.1863	C	-4.9707	2.1986	-1.1894
C	-5.0390	-2.1086	1.2299	C	-5.0529	-2.1041	1.2314
C	-5.8017	-3.3059	1.3883	C	-5.8188	-3.3003	1.3849
C	-6.8913	-3.5938	0.5029	C	-6.8980	-3.5900	0.4873
C	-7.2124	-2.6729	-0.5474	C	-7.2058	-2.6723	-0.5700

**C**	-6.4518	-1.4758	-0.7180	**C**	-6.4418	-1.4763	-0.7354
**H**	7.5643	1.9868	1.0571	**H**	7.5672	2.0062	1.0203
**H**	10.0692	1.7878	0.9715	**H**	10.0702	1.8058	0.9361
**H**	9.9201	-1.9513	-0.9823	**H**	9.9196	-1.9701	-0.9494
**H**	7.3871	-1.9686	-1.0100	**H**	7.3888	-1.9880	-0.9746
**H**	-0.5720	-2.0541	-1.1449	**H**	-0.5723	-2.0696	-1.1173
**H**	-3.0566	-1.9644	-1.0861	**H**	-3.0548	-1.9791	-1.0597
**H**	-2.8926	1.9383	1.0362	**H**	-2.8905	1.9518	1.0132
**H**	-0.3881	1.8585	0.9955	**H**	-0.3884	1.8716	0.9731
**H**	-6.5913	0.9426	1.6456	**H**	-6.5706	0.9495	1.6642
**H**	-7.8357	3.1468	1.4042	**H**	-7.8179	3.1531	1.4283
**H**	-7.2441	4.7587	-0.4926	**H**	-7.2462	4.7583	-0.4804
**H**	-5.3984	4.1363	-2.1523	**H**	-5.4186	4.1296	-2.1580
**H**	-4.1638	1.9251	-1.9213	**H**	-4.1824	1.9185	-1.9326
**H**	-4.2006	-1.8763	1.9338	**H**	-4.2233	-1.8697	1.9449
**H**	-5.5506	-4.0124	2.2200	**H**	-5.5785	-4.0040	2.2221
**H**	-7.4893	-4.5314	0.6322	**H**	-7.4985	-4.5265	0.6127
**H**	-8.0572	-2.8928	-1.2488	**H**	-8.0425	-2.8937	-1.2805
**H**	-6.6984	-0.7639	-1.5452	**H**	-6.6774	-0.7671	-1.5681

Ph2N-TTz-CHO in emission states

toluene				chloroform			
**C**	-3.8402	-0.0043	-0.0200	**C**	-3.8396	-0.0044	-0.0147
**C**	-3.0394	1.0711	0.5469	**C**	-3.0374	1.0856	0.5255
**C**	-1.6315	1.0211	0.5155	**C**	-1.6303	1.0334	0.4981
**C**	-0.9246	-0.1105	-0.0901	**C**	-0.9217	-0.1144	-0.0769
**C**	-1.7391	-1.1818	-0.6581	**C**	-1.7372	-1.2004	-0.6177
**C**	-3.1500	-1.1300	-0.6235	**C**	-3.1469	-1.1475	-0.5867
**C**	0.5311	-0.1380	-0.1050	**C**	0.5314	-0.1436	-0.0907
**N**	1.3175	0.8503	0.4084	**N**	1.3213	0.8614	0.3915
**C**	2.6183	0.5593	0.2633	**C**	2.6199	0.5657	0.2548
**C**	2.9902	-0.6892	-0.3797	**C**	2.9919	-0.7044	-0.3495
**S**	1.4970	-1.5666	-0.8393	**S**	1.4975	-1.5952	-0.7797
**S**	4.1203	1.4366	0.7272	**S**	4.1225	1.4577	0.6883
**C**	5.0787	-0.0022	-0.0095	**C**	5.0818	-0.0034	-0.0032
**N**	4.2927	-0.9905	-0.5253	**N**	4.2922	-1.0093	-0.4870
**C**	6.5391	-0.0346	-0.0139	**C**	6.5388	-0.0364	-0.0104
**C**	7.3429	1.0462	0.5537	**C**	7.3459	1.0630	0.5205
**C**	8.7556	0.9867	0.5392	**C**	8.7579	1.0038	0.5020
**C**	9.4422	-0.1489	-0.0397	**C**	9.4453	-0.1493	-0.0443
**C**	8.6545	-1.2221	-0.6051	**C**	8.6548	-1.2416	-0.5721
**C**	7.2396	-1.1782	-0.5987	**C**	7.2406	-1.1986	-0.5614
**C**	10.9459	-0.2265	-0.0593	**C**	10.9455	-0.2277	-0.0700
**O**	11.7100	0.6272	0.3942	**O**	11.7151	0.6412	0.3528
**N**	-5.2602	0.0445	0.0204	**N**	-5.2555	0.0464	0.0194
**C**	-6.0367	-1.1735	0.1953	**C**	-6.0384	-1.1718	0.1808
**C**	-5.9615	1.3119	-0.1165	**C**	-5.9581	1.3159	-0.1115
**C**	-5.7124	-2.0884	1.2556	**C**	-5.7426	-2.0793	1.2547
**C**	-6.4796	-3.2810	1.4268	**C**	-6.5165	-3.2697	1.4140
**C**	-7.5776	-3.5687	0.5524	**C**	-7.5925	-3.5604	0.5133
**C**	-7.9019	-2.6525	-0.5003	**C**	-7.8885	-2.6500	-0.5531
**C**	-7.1358	-1.4608	-0.6844	**C**	-7.1152	-1.4605	-0.7247
**C**	-6.9943	1.6687	0.8167	**C**	-6.9781	1.6742	0.8341
**C**	-7.6869	2.9094	0.6713	**C**	-7.6735	2.9145	0.6937

**C**	-7.3542	3.8062	-0.3956	**C**	-7.3554	3.8073	-0.3814
**C**	-6.3225	3.4491	-1.3233	**C**	-6.3366	3.4470	-1.3223
**C**	-5.6295	2.2071	-1.1910	**C**	-5.6413	2.2054	-1.1945
**H**	-3.5469	1.9406	1.0331	**H**	-3.5436	1.9675	0.9893
**H**	-1.0378	1.8464	0.9773	**H**	-1.0389	1.8707	0.9407
**H**	-1.2528	-2.0601	-1.1531	**H**	-1.2518	-2.0914	-1.0901
**H**	-3.7409	-1.9601	-1.0831	**H**	-3.7359	-1.9889	-1.0269
**H**	6.8459	1.9401	1.0102	**H**	6.8509	1.9705	0.9512
**H**	9.3576	1.8217	0.9776	**H**	9.3583	1.8541	0.9123
**H**	9.1731	-2.1085	-1.0581	**H**	9.1719	-2.1416	-0.9987
**H**	6.6459	-2.0153	-1.0392	**H**	6.6480	-2.0508	-0.9732
**H**	11.3700	-1.1759	-0.5456	**H**	11.3674	-1.1905	-0.5280
**H**	-4.8652	-1.8573	1.9498	**H**	-4.9133	-1.8450	1.9692
**H**	-6.2248	-3.9846	2.2603	**H**	-6.2848	-3.9684	2.2582
**H**	-8.1794	-4.5027	0.6919	**H**	-8.1998	-4.4922	0.6434
**H**	-8.7540	-2.8716	-1.1935	**H**	-8.7236	-2.8715	-1.2659
**H**	-7.3866	-0.7509	-1.5126	**H**	-7.3433	-0.7551	-1.5631
**H**	-7.2503	0.9742	1.6562	**H**	-7.2227	0.9825	1.6793
**H**	-8.4870	3.1826	1.4061	**H**	-8.4641	3.1898	1.4378
**H**	-7.8981	4.7790	-0.5044	**H**	-7.9014	4.7793	-0.4867
**H**	-6.0621	4.1377	-2.1675	**H**	-6.0888	4.1319	-2.1732
**H**	-4.8344	1.9231	-1.9261	**H**	-4.8576	1.9179	-1.9403

Ph2N-TTz-CHO in emission states

DCM				methanol			
C	-3.8390	-0.0049	-0.0129	C	-3.8384	-0.0063	-0.0111
C	-3.0359	1.0930	0.5121	C	-3.0338	1.0990	0.4984
C	-1.6293	1.0395	0.4868	C	-1.6279	1.0435	0.4756
C	-0.9200	-0.1170	-0.0713	C	-0.9182	-0.1221	-0.0646
C	-1.7363	-1.2106	-0.5974	C	-1.7357	-1.2229	-0.5754
C	-3.1453	-1.1571	-0.5679	C	-3.1440	-1.1681	-0.5481
C	0.5316	-0.1473	-0.0843	C	0.5317	-0.1539	-0.0761
N	1.3232	0.8658	0.3826	N	1.3248	0.8677	0.3748
C	2.6205	0.5684	0.2499	C	2.6209	0.5688	0.2464
C	2.9930	-0.7120	-0.3357	C	2.9941	-0.7219	-0.3192
S	1.4981	-1.6091	-0.7515	S	1.4991	-1.6260	-0.7194
S	4.1232	1.4673	0.6691	S	4.1232	1.4754	0.6503
C	5.0834	-0.0036	-0.0006	C	5.0848	-0.0051	0.0032
N	4.2920	-1.0180	-0.4691	N	4.2920	-1.0286	-0.4487
C	6.5383	-0.0370	-0.0088	C	6.5377	-0.0380	-0.0069
C	7.3471	1.0707	0.5056	C	7.3477	1.0784	0.4902
C	8.7586	1.0116	0.4855	C	8.7587	1.0203	0.4675
C	9.4469	-0.1492	-0.0459	C	9.4485	-0.1475	-0.0490
C	8.6552	-1.2499	-0.5566	C	8.6559	-1.2570	-0.5417
C	7.2415	-1.2074	-0.5442	C	7.2426	-1.2160	-0.5266
C	10.9451	-0.2281	-0.0743	C	10.9447	-0.2258	-0.0810
O	11.7176	0.6477	0.3345	O	11.7195	0.6578	0.3120
N	-5.2525	0.0471	0.0188	N	-5.2493	0.0479	0.0175
C	-6.0392	-1.1708	0.1739	C	-6.0412	-1.1689	0.1659
C	-5.9558	1.3178	-0.1088	C	-5.9520	1.3206	-0.1068
C	-5.7595	-2.0731	1.2560	C	-5.7800	-2.0658	1.2565
C	-6.5376	-3.2618	1.4101	C	-6.5638	-3.2518	1.4049
C	-7.6014	-3.5548	0.4955	C	-7.6145	-3.5463	0.4756
C	-7.8812	-2.6488	-0.5792	C	-7.8759	-2.6447	-0.6076
C	-7.1035	-1.4610	-0.7452	C	-7.0924	-1.4596	-0.7674
C	-6.9647	1.6795	0.8468	C	-6.9466	1.6884	0.8609
C	-7.6617	2.9195	0.7090	C	-7.6439	2.9290	0.7257

**C**	-7.3558	3.8073	-0.3739	**C**	-7.3518	3.8098	-0.3667
**C**	-6.3479	3.4428	-1.3252	**C**	-6.3578	3.4385	-1.3301
**C**	-5.6512	2.2015	-1.1995	**C**	-5.6610	2.1968	-1.2068
**H**	-3.5413	1.9814	0.9637	**H**	-3.5381	1.9944	0.9370
**H**	-1.0390	1.8832	0.9184	**H**	-1.0383	1.8935	0.8958
**H**	-1.2515	-2.1081	-1.0576	**H**	-1.2517	-2.1271	-1.0229
**H**	-3.7334	-2.0043	-0.9974	**H**	-3.7315	-2.0208	-0.9668
**H**	6.8529	1.9840	0.9245	**H**	6.8540	1.9971	0.8974
**H**	9.3577	1.8688	0.8833	**H**	9.3562	1.8847	0.8521
**H**	9.1717	-2.1557	-0.9710	**H**	9.1722	-2.1683	-0.9441
**H**	6.6498	-2.0664	-0.9429	**H**	6.6522	-2.0820	-0.9117
**H**	11.3662	-1.1964	-0.5191	**H**	11.3655	-1.1997	-0.5119
**H**	-4.9404	-1.8364	1.9812	**H**	-4.9716	-1.8272	1.9931
**H**	-6.3190	-3.9568	2.2607	**H**	-6.3599	-3.9431	2.2621
**H**	-8.2121	-4.4849	0.6215	**H**	-8.2296	-4.4741	0.5969
**H**	-8.7067	-2.8722	-1.3024	**H**	-8.6911	-2.8694	-1.3420
**H**	-7.3183	-0.7591	-1.5900	**H**	-7.2921	-0.7613	-1.6190
**H**	-7.1994	0.9916	1.6980	**H**	-7.1694	1.0060	1.7195
**H**	-8.4438	3.1983	1.4607	**H**	-8.4150	3.2133	1.4866
**H**	-7.9030	4.7788	-0.4775	**H**	-7.8992	4.7814	-0.4684
**H**	-6.1102	4.1236	-2.1822	**H**	-6.1313	4.1138	-2.1945
**H**	-4.8768	1.9099	-1.9535	**H**	-4.8982	1.8990	-1.9700

Ph2N-TTz-COOH in emission states

toluene				chloroform			
**C**	0.1757	-0.1129	-0.0872	**C**	0.1754	-0.1159	-0.0822
**N**	0.9554	0.8804	0.4353	**N**	0.9586	0.8930	0.4120
**C**	2.2570	0.5987	0.2914	**C**	2.2581	0.6067	0.2765
**C**	2.6405	-0.6448	-0.3594	**C**	2.6421	-0.6570	-0.3383
**S**	1.1547	-1.5321	-0.8286	**S**	1.1552	-1.5566	-0.7817
**S**	3.7525	1.4829	0.7655	**S**	3.7543	1.5047	0.7236
**C**	4.7229	0.0560	0.0217	**C**	4.7257	0.0567	0.0228
**N**	3.9455	-0.9336	-0.5024	**N**	3.9453	-0.9494	-0.4726
**C**	6.1854	0.0357	0.0199	**C**	6.1854	0.0354	0.0205
**C**	-1.2777	-0.0961	-0.0779	**C**	-1.2754	-0.0977	-0.0732
**C**	6.9793	1.1114	0.6015	**C**	6.9821	1.1290	0.5680
**C**	8.3972	1.0612	0.5861	**C**	8.3994	1.0781	0.5522
**C**	8.3113	-1.1311	-0.5884	**C**	8.3125	-1.1497	-0.5542
**C**	6.8970	-1.0933	-0.5791	**C**	6.8986	-1.1122	-0.5445
**C**	-2.0834	-1.1746	-0.6495	**C**	-2.0825	-1.1894	-0.6203
**C**	-3.4948	-1.1323	-0.6204	**C**	-3.4928	-1.1467	-0.5926
**C**	-4.1956	-0.0111	-0.0204	**C**	-4.1958	-0.0108	-0.0188
**C**	-3.4051	1.0707	0.5498	**C**	-3.4037	1.0845	0.5265
**C**	-1.9968	1.0315	0.5251	**C**	-1.9962	1.0441	0.5034
**N**	-5.6174	0.0278	0.0139	**N**	-5.6137	0.0288	0.0128
**C**	-6.3848	-1.1939	0.2011	**C**	-6.3854	-1.1938	0.1900
**C**	-6.3262	1.2885	-0.1433	**C**	-6.3248	1.2911	-0.1362
**C**	-7.3699	1.6493	0.7769	**C**	-7.3548	1.6522	0.7983
**C**	-8.0694	2.8838	0.6115	**C**	-8.0576	2.8864	0.6403
**C**	-7.7328	3.7715	-0.4623	**C**	-7.7372	3.7713	-0.4410
**C**	-6.6898	3.4111	-1.3763	**C**	-6.7079	3.4091	-1.3701
**C**	-5.9898	2.1749	-1.2242	**C**	-6.0049	2.1734	-1.2249
**C**	-6.0520	-2.0973	1.2691	**C**	-6.0732	-2.0915	1.2682
**C**	-6.8100	-3.2943	1.4522	**C**	-6.8357	-3.2875	1.4426
**C**	-7.9074	-3.5984	0.5821	**C**	-7.9167	-3.5943	0.5528
**C**	-8.2398	-2.6941	-0.4788	**C**	-8.2288	-2.6943	-0.5182
**C**	-7.4828	-1.4983	-0.6750	**C**	-7.4670	-1.4995	-0.7051

**H**	6.4798	1.9965	1.0719	**H**	6.4842	2.0285	1.0121
**H**	8.9796	1.8998	1.0403	**H**	8.9807	1.9312	0.9800
**H**	8.8424	-2.0003	-1.0504	**H**	8.8408	-2.0339	-0.9904
**H**	6.3106	-1.9293	-1.0320	**H**	6.3138	-1.9629	-0.9712
**H**	-1.5886	-2.0499	-1.1418	**H**	-1.5887	-2.0754	-1.0940
**H**	-4.0785	-1.9673	-1.0814	**H**	-4.0750	-1.9917	-1.0361
**H**	-3.9219	1.9371	1.0327	**H**	-3.9191	1.9615	0.9907
**H**	-1.4110	1.8615	0.9890	**H**	-1.4124	1.8851	0.9498
**H**	-7.6290	0.9625	1.6222	**H**	-7.6011	0.9673	1.6488
**H**	-8.8781	3.1597	1.3363	**H**	-8.8560	3.1635	1.3758
**H**	-8.2822	4.7397	-0.5867	**H**	-8.2891	4.7388	-0.5599
**H**	-6.4257	4.0924	-2.2257	**H**	-6.4575	4.0878	-2.2257
**H**	-5.1858	1.8883	-1.9488	**H**	-5.2129	1.8844	-1.9617
**H**	-5.2051	-1.8538	1.9598	**H**	-5.2401	-1.8450	1.9744
**H**	-6.5485	-3.9887	2.2917	**H**	-6.5910	-3.9780	2.2902
**H**	-8.5021	-4.5359	0.7310	**H**	-8.5151	-4.5306	0.6947
**H**	-9.0912	-2.9262	-1.1692	**H**	-9.0675	-2.9285	-1.2231
**H**	-7.7399	-0.7981	-1.5099	**H**	-7.7077	-0.8028	-1.5476
**C**	9.0888	-0.0585	-0.0074	**C**	9.0921	-0.0594	-0.0078
**C**	10.6017	-0.1519	-0.0433	**C**	10.6037	-0.1519	-0.0425
**O**	11.2371	-1.0823	-0.5311	**O**	11.2399	-1.0975	-0.5045
**O**	11.2236	0.9240	0.5333	**O**	11.2255	0.9391	0.5007
**H**	12.1967	0.7757	0.4635	**H**	12.1997	0.7935	0.4362

Ph2N-TTz-COOH in emission states

DCM				methanol			
**C**	0.1752	-0.1191	-0.0749	**C**	0.1749	-0.1224	-0.0703
**N**	0.9604	0.8968	0.4062	**N**	0.9618	0.9010	0.3968
**C**	2.2586	0.6088	0.2741	**C**	2.2588	0.6115	0.2685
**C**	2.6429	-0.6639	-0.3246	**C**	2.6436	-0.6706	-0.3126
**S**	1.1554	-1.5692	-0.7552	**S**	1.1559	-1.5819	-0.7302
**S**	3.7552	1.5130	0.7085	**S**	3.7556	1.5222	0.6896
**C**	4.7272	0.0565	0.0260	**C**	4.7284	0.0568	0.0275
**N**	3.9449	-0.9573	-0.4560	**N**	3.9447	-0.9649	-0.4402
**C**	6.1851	0.0349	0.0220	**C**	6.1847	0.0349	0.0228
**C**	-1.2742	-0.0998	-0.0668	**C**	-1.2730	-0.1020	-0.0629
**C**	6.9838	1.1356	0.5552	**C**	6.9851	1.1436	0.5393
**C**	8.4008	1.0848	0.5370	**C**	8.4018	1.0927	0.5200
**C**	8.3128	-1.1565	-0.5424	**C**	8.3132	-1.1645	-0.5262
**C**	6.8991	-1.1196	-0.5305	**C**	6.8997	-1.1281	-0.5132
**C**	-2.0820	-1.1979	-0.6015	**C**	-2.0817	-1.2063	-0.5853
**C**	-3.4917	-1.1547	-0.5754	**C**	-3.4907	-1.1625	-0.5603
**C**	-4.1957	-0.0107	-0.0164	**C**	-4.1956	-0.0111	-0.0152
**C**	-3.4029	1.0912	0.5164	**C**	-3.4018	1.0972	0.5050
**C**	-1.9958	1.0496	0.4955	**C**	-1.9952	1.0545	0.4854
**N**	-5.6114	0.0299	0.0123	**N**	-5.6091	0.0304	0.0116
**C**	-6.3861	-1.1929	0.1833	**C**	-6.3869	-1.1924	0.1770
**C**	-6.3232	1.2931	-0.1343	**C**	-6.3215	1.2948	-0.1313
**C**	-7.3468	1.6556	0.8063	**C**	-7.3352	1.6601	0.8185
**C**	-8.0511	2.8895	0.6501	**C**	-8.0406	2.8941	0.6654
**C**	-7.7382	3.7717	-0.4358	**C**	-7.7381	3.7724	-0.4267
**C**	-6.7154	3.4072	-1.3715	**C**	-6.7250	3.4045	-1.3718
**C**	-6.0112	2.1718	-1.2278	**C**	-6.0198	2.1690	-1.2308
**C**	-6.0887	-2.0856	1.2695	**C**	-6.1035	-2.0805	1.2703
**C**	-6.8543	-3.2805	1.4387	**C**	-6.8723	-3.2743	1.4347
**C**	-7.9234	-3.5904	0.5355	**C**	-7.9304	-3.5865	0.5192
**C**	-8.2206	-2.6946	-0.5435	**C**	-8.2138	-2.6945	-0.5667
**C**	-7.4556	-1.5008	-0.7250	**C**	-7.4453	-1.5017	-0.7431

**H**	6.4872	2.0402	0.9899	**H**	6.4894	2.0545	0.9615
**H**	8.9817	1.9437	0.9538	**H**	8.9821	1.9584	0.9234
**H**	8.8393	-2.0463	-0.9692	**H**	8.8382	-2.0612	-0.9405
**H**	6.3152	-1.9764	-0.9460	**H**	6.3167	-1.9919	-0.9155
**H**	-1.5888	-2.0896	-1.0649	**H**	-1.5891	-2.1031	-1.0392
**H**	-4.0730	-2.0044	-1.0103	**H**	-4.0713	-2.0167	-0.9868
**H**	-3.9176	1.9737	0.9703	**H**	-3.9157	1.9849	0.9491
**H**	-1.4133	1.8960	0.9331	**H**	-1.4137	1.9062	0.9141
**H**	-7.5871	0.9729	1.6604	**H**	-7.5667	0.9805	1.6774
**H**	-8.8445	3.1683	1.3902	**H**	-8.8265	3.1757	1.4124
**H**	-8.2911	4.7386	-0.5536	**H**	-8.2919	4.7391	-0.5423
**H**	-6.4715	4.0833	-2.2309	**H**	-6.4898	4.0774	-2.2360
**H**	-5.2252	1.8804	-1.9700	**H**	-5.2422	1.8742	-1.9805
**H**	-5.2655	-1.8360	1.9862	**H**	-5.2895	-1.8284	1.9965
**H**	-6.6216	-3.9674	2.2926	**H**	-6.6509	-3.9580	2.2941
**H**	-8.5242	-4.5256	0.6733	**H**	-8.5337	-4.5206	0.6532
**H**	-9.0500	-2.9313	-1.2585	**H**	-9.0346	-2.9331	-1.2909
**H**	-7.6841	-0.8076	-1.5737	**H**	-7.6623	-0.8116	-1.5974
**C**	9.0940	-0.0592	-0.0110	**C**	9.0956	-0.0591	-0.0122
**C**	10.6046	-0.1504	-0.0479	**C**	10.6054	-0.1495	-0.0498
**O**	11.2410	-1.1015	-0.5015	**O**	11.2420	-1.1077	-0.4913
**O**	11.2269	0.9462	0.4812	**O**	11.2279	0.9538	0.4623
**H**	12.2017	0.8029	0.4172	**H**	12.2032	0.8122	0.3999
